# Heat shock proteins expressed in the marsupial Tasmanian devil are potential antigenic candidates in a vaccine against devil facial tumour disease

**DOI:** 10.1371/journal.pone.0196469

**Published:** 2018-04-27

**Authors:** Cesar Tovar, Amanda L. Patchett, Vitna Kim, Richard Wilson, Jocelyn Darby, A. Bruce Lyons, Gregory M. Woods

**Affiliations:** 1 Menzies Institute for Medical Research, University of Tasmania, Hobart, Tasmania, Australia; 2 Central Science Laboratory, University of Tasmania, Hobart, Tasmania, Australia; 3 School of Medicine, University of Tasmania, Hobart, Tasmania, Australia; Technische Universitat Munchen, GERMANY

## Abstract

The Tasmanian devil (*Sarcophilus harrisii)*, the largest extant carnivorous marsupial and endemic to Tasmania, is at the verge of extinction due to the emergence of a transmissible cancer known as devil facial tumour disease (DFTD). DFTD has spread over the distribution range of the species and has been responsible for a severe decline in the global devil population. To protect the Tasmanian devil from extinction in the wild, our group has focused on the development of a prophylactic vaccine. Although this work has shown that vaccine preparations using whole DFTD tumour cells supplemented with adjuvants can induce anti-DFTD immune responses, alternative strategies that induce stronger and more specific immune responses are required. In humans, heat shock proteins (HSPs) derived from tumour cells have been used instead of whole-tumour cell preparations as a source of antigens for cancer immunotherapy. As HSPs have not been studied in the Tasmanian devil, this study presents the first characterisation of HSPs in this marsupial and evaluates the suitability of these proteins as antigenic components for the enhancement of a DFTD vaccine. We show that tissues and cancer cells from the Tasmanian devil express constitutive and inducible HSP. Additionally, this study suggests that HSP derived from DFTD cancer cells are immunogenic supporting the future development of a HSP-based vaccine against DFTD.

## Introduction

The carnivorous marsupial Tasmanian devil (*Sarcophilus harrisii*) is at risk of extinction due to a transmissible cancer known as devil facial tumour disease (DFTD). This disease emerged in northeast Tasmania in 1996 and has spread to almost the entire wild devil population. DFTD is a unique cancer as the tumour cells themselves are the infectious agent transmitted between unrelated devils through biting. The disease is characterised by the appearance of large, aggressive tumours around the face and neck and most affected animals die from starvation and organ failure due to metastases. To colonise a new host, DFTD cells evade allorecognition by downregulating molecules of the major histocompatibility complex class I (MHC-I) and antigen presenting machinery [1]. However, the existence of other strategies of tumour escape is largely unknown. A second transmissible cancer was identified in Tasmanian devils in 2014 and was referred to as DFT2 to distinguish from the first DFTD (discovered in 1996, thereby denoted DFT1). This manuscript relates only to DFT1.

To protect the Tasmanian devil from extinction in the wild our group has focused on the development of a prophylactic DFTD vaccine. We have shown that vaccine preparations using whole DFTD tumours cells supplemented with adjuvants can induce humoral responses [2]. Furthermore, we recently showed that DFTD cancer cells can be targeted *in vivo* by the Tasmanian devil’s immune system [3]. These studies demonstrated that Tasmanian devils are able to mount specific humoral and cellular responses leading to the regression of established DFTD tumours. The findings also highlighted the feasibility of developing a vaccine. Our approach used a combination of irradiated DFTD cells or whole cell lysates as antigen, plus adjuvants. While this vaccine, elicited specific humoral responses, this approach was not completely protective and tumours developed after challenge with live DFTD cells. As this whole-tumour cell vaccine showed limited efficacy, alternative strategies that induce stronger and more specific immune responses are required. An ideal vaccine would be highly immunogenic and enriched with specific DFTD antigens.

Heat shock proteins (HSPs) have become an attractive source of antigens for cancer immunotherapy as an alternative to whole-tumour cell preparations. These proteins are among the most abundant and ubiquitous intracellular proteins and are highly conserved across species [4]. HSPs are molecular chaperones involved in numerous cellular processes including protein folding, transport, assembly and peptide trafficking in antigen presentation. Most HSPs are expressed constitutively in all cells during normal growth conditions, but their expression is upregulated under environmental stressors that are unfavourable for protein folding and association [5]. The use of HSPs for vaccination is based on studies in humans and other animal models showing that immunisation with HSPs elicits potent anti-tumour effects [6–8]. The immunogenicity of HSPs relates to their capacity to carry antigenic peptides from the tumour cells from which the HSPs were isolated [9]. The response is mediated by antigen presenting cells (APCs) that take up the HSP-peptide complexes and present the antigenic peptides to CD8^+^ and CD4^+^ T cells [10]. The interaction of HSP–peptide complexes with APC receptors also induces innate immune responses. These responses include the maturation of dendritic cells and the release of cytokines and chemokines by APC and T cells [11].

In the clinical setting, the use of autologous tumour-derived HSPs as an anti-cancer vaccine has been widely studied. HSP-peptide complexes (HSPPC) can be purified from solid tumours and have been safely tested in a variety of cancer patients. Thus, HSPs derived from tumours can be used as tumour-specific vaccines. Members of the HSP90 family have been particularly used for this approach. Phase I, II and III trials of autologous HSPPC-gp96 vaccination in human melanoma, renal carcinoma, glioblastoma and colon carcinoma have demonstrated that immunisation elicits tumour-specific immune responses. No relevant toxicity has been observed. Overall survival and disease-free survival has improved in some patients with an immune response. [12–16]. In an alternative approach, the use of HSPs alone (i.e. peptide-independent) has an immunomodulatory activity within the tumour microenvironment. This is because HSPs function as adjuvants that enhance innate and adaptive immune responses. HSPs can move to the extracellular space in soluble form or within exosomes and bind to specific receptors in a number of cells including natural killer (NK) cells, dendritic cells (DC) macrophages, peripheral blood monocytes, and B cells [17, 18]. Therefore, HSPs induce expression of costimulatory molecules, maturation of DC, secretion of proinflammatory cytokines and activation and migration of NK cells [19–21]. Due to these immunomodulatory activities, HSP70 has been used as a treatment to enhance specific anti-tumour immune responses. In preclinical studies and clinical trials involving brain tumours, hepatocellular carcinoma, colon or lung cancers, intratumoural injection of HSP70, upregulation of HSP70 within the tumour or the use of selective HSP70 peptides for ex vivo stimulation of immune cells, stimulated potent anti-tumour immune responses [22–25].

HSPs have not been studied in the Tasmanian devil. Consequently, this study presents the first characterisation of HSPs in this marsupial and evaluates the suitability of these proteins as components for the enhancement of a DFTD vaccine. Initially, we used quantitative reverse transcriptase polymerase chain reaction (qRT-PCR) and protein immunoblot to examine the expression of a selected range of HSPs in in normal Tasmanian devil tissue, primary DFTD tumours and DFTD cell lines. We also used these techniques to investigate the occurrence of inducible HSPs in DFTD cells *in vitro*, by exposing the cells to environmental stressors such as heat shock and radiation. Finally, we used an immunoproteomic approach to investigate whether circulating antibodies against HSPs were present in the serum of devils previously immunised with whole-DFTD cell vaccine preparations.

## Materials and methods

### Tissue samples and cell lines

This study was carried out in strict accordance with the recommendations in the University of Tasmania guidelines. All animal procedures were approved by the University of Tasmania Animal Ethics Committee under A009215, A0012513 and A0014976. Tasmanian devil tissue samples and primary DFTD tumours were supplied by the Mount Pleasant Laboratories from the Tasmanian Department of Primary Industries, Parks, Water and Environment (DPIPWE). For this study, we used samples from three DFTD primary tumours (TD1- TD592, -TD2-TD284 and TD3- TD285 and) and skin tissue samples from three animals (TD502, TD518 and TD579). DFT1 cell lines were provided by A.-M. Pearse and K. Swift of the DPIPWE. These cell lines were previously established from DFT1 biopsies obtained under research authorities 33/2004-2005 and 24/2006-2008 issued by the Animal Ethics Committee of the DPIWE [26]. Three DFTD cells lines were used: strain 2 (1426), strain 3 (C5065) and strain 4 (4906). The cell lines were cultured in RPMI culture medium (Thermo Fisher Scientific, Waltham, USA) containing 10% foetal calf serum (GIBCO, New York, USA), 1% GlutaMAX^TM^ (GIBCO, New York, USA) and 1% Antibiotic Antimycotic (GIBCO, New York, USA) (complete culture medium) and kept at 35 ^o^C, 5% CO_2_ in a humidified incubator.

### RNA extraction and quantitative reverse transcriptase polymerase chain reaction (qRT-PCR)

Tissue samples were homogenised in TriReagent (Sigma-Aldrich, St Louis, USA) with a Mini Beadbeater-24 (BioSpec Products,Bartlesville, USA) and 2.0 mm Zirconia Beads (BioSpec Products, Bartlesville, USA). Cell lines were lysed in TriReagent without homogenisation. RNA extraction and cDNA preparation was performed as previously described [27]. Primer sequences were designed for target genes identified in the Tasmanian devil reference genome Devil7.0 assembly GCA_000189315.1, using the NCBI and Ensembl PrimerBLAST tool (S1 Table). Primers were synthesised by GeneWorks (Adelaide, Australia). qRT-PCR was performed using the LightCycler® 480 (Roche, Indianapolis, USA) as previously described [27]. Expression of ribosomal protein 18 (*RPS18*) was used as reference gene. All analyses included no-template controls and no-RT controls. Reaction efficiency was validated using standard curves, and the comparative Ct method was used for calculation of expression fold change [28]. Statistical significance was calculated from log2-converted values using a repeated measures one-way ANOVA and a Dunnett's multiple comparisons test. GraphPad Prism Version 6.07 was used for statistical analysis and to generate the graphs. Statistical significance relative to the untreated control is reported as multiplicity adjusted P value: * *p*<0.05, ** *p*<0.01 and *** *p*<0.001.

### Protein extraction and western blot

Total cell protein from DFTD cell lines was extracted with 1 ml of RIPA buffer (Thermo Scientific, Rockford, IL) containing 10 μl of HALT™ protease inhibitor cocktail (Thermo Scientific, Rock-ford, USA) for approximately 40 mg of wet cell pellet. The suspension was sonicated three times for 15 seconds with 50% pulse and then centrifuged at 14,000 X *g* for 15 minutes. The supernatant was transferred to a new tube for protein quantification using the EZQ® Protein Quantitation kit (Molecular Probes, Eugen, USA) according to the manufacturer’s instructions. For total protein extraction from devil tissues, samples were homogenised with RIPA buffer containing protease inhibitors using a Mini Beadbeater-24 (BioSpec Products, Bartlesville, USA) and 2.0 mm Zirconia Beads (BioSpec Products, Bartlesville, USA). The protein solution was sonicated, centrifuged and protein quantification determined as indicated above.

For protein electrophoresis 10 μg of protein sample was added to Bolt™ LDS Sample Buffer (4X) (LifeTechnologies, Carlsbad, USA) and Bolt™ Sample Reducing Agent (10X) with the remaining volume made up by Milli-Q water to 20 μl. The samples were centrifuged and heated at 70 ^o^C for 10 min. Samples and a molecular weight marker were loaded on Bolt®4–12% Bis-Tris Plus mini-gels. The gels were run at 165 V constant for 50 min using Bolt™ MES SDS running buffer and the Bolt® mini gel tank. The iBlot® and iBind™ Western Blotting systems were used for protein electroblotting to nitrocellulose (20 V for 7.5 min) and subsequent detection according to the manufacturer’s instructions (Life Technologies, Carlsbad, USA). Total incubation time with primary and secondary antibodies (S2 Table) was 3 hours. Immobilon™ Western HRP substrate (Merck Millipore, Billerica, USA) was used for enhanced chemiluminescent (ECL) detection for 5 minutes followed by imaging using an Amersham™ Imager 600.

Protein expression levels were assessed using the densitometry data acquired from the images of the western blots. A loading amount of 10 μg of total protein was determined to be optimal for detecting changes in protein expression for each of the antibodies used (see S1 Fig). Each sample was run in duplicate. A second gel was run in parallel to determine total protein using SYPRO® Ruby Protein Gel Stain (Life Technologies, Eugen, USA) following the manufacturer’s instructions. Fluorescent images were taken using the Amersham™ Imager 600. ImageQuant TL 8.1 software was used for densitometry analysis of the fluorescent gels and chemiluminescent blots. Background subtraction was determined using the rolling ball tool and automatic detection of band edges. We use total protein as a loading control. Thus, the band density of each sample in the chemiluminescent blot was normalised against the density of the total protein (whole lane) in the fluorescent gel. Images of the blots and the band analysis are shown in S2 Fig. Final protein expression for each sample was calculated relative to the control (untreated) sample. Statistical significance was calculated using a one-way ANOVA of log transformed differential expression data. GraphPad Prism Version 6.07 was used for statistical analysis and to generate the graphs. Statistical significance relative to the untreated control is reported as multiplicity adjusted P value: * *p*<0.05, ** *p*<0.01 and *** *p*<0.001.

### Heat shock treatment

C5065 DFTD cells were plated at 2x10^5^ cells/well in 6 well trays and grown overnight in complete culture medium. Duplicate trays were placed in a 42°C water bath for 30 minutes then returned to the 35°C incubator. Samples were collected for RNA and protein extraction at 0, 4, 8 and 24 hours post heat shock. For statistical analysis, the duplicates were treated as multiple observations of the same test condition.

For cell viability analyses after heat shock, C5065 DFTD cells were treated as indicated above at 35°C, 42°C or 45°C for 30, 60 or 120 minutes. In a separate experiment, the cells were treated under the same conditions but allowed to recover for 24 hours under normal culture conditions in a humidified incubator at 35 ^o^C. After the treatments, the cells were harvested from the wells, washed twice in washing buffer (PBS buffer containing 0.5% bovine serum albumin and 0.02% sodium azide) and resuspended in 350 μl of washing buffer plus 2 μg/ml propidium iodide (Sigma-Aldrich, Saint Louis, USA). Cell viability was evaluated by flow cytometry using a CyAn flow cytometer (Beckman Coulter, Brea, USA). The gating strategy used for discriminating and quantifying viable and non-viable cells is shown in S3 Fig.

### Radiation treatment

C5065 DFTD cells were suspended in complete culture medium and placed in 1.8 ml cryovials and irradiated with 20 Gy of gamma radiation using a Varian Clinac 23-EX linear accelerator–Varian Medical Systems Inc. Post irradiation cells were returned to normal culture conditions at 35°C for 24 hours before samples were collected for RNA and protein extraction. Control cells remained untreated at 35°C in the incubator.

### Immunoproteome approach to study tumour antigens

#### Two-dimensional electrophoresis (2DE)

2DE was performed using the ZOOM® IPGRunner™ System and the Xcell SureLock™ mini cell (Invitrogen, Carlsbad, USA) following the instructions of the manufacturer. A summary of the protocol is presented here:

Sample preparation: A 950 μl aliquot of chilled lysis buffer containing ZOOM® 2D protein solubilizer (Invitrogen, Carlsbad, USA), 1 M tris-base, HALT™ protease inhibitor cocktail and 2 M DTT (Invitrogen, Carlsbad, USA), was added to 50 μl of pelleted C5065 DFTD cells and the sample sonicated. The lysate was then alkylated with N,N-dimethylacrylamide (DMA) (Sigma-Aldrich, St Louis, USA) and excess DMA quenched with 2 M DTT, centrifuged, and the supernatant transferred to a new microcentrifuge tube. A 12 μl aliquot of the lysate containing approximately 15 μg of protein was diluted with sample rehydration buffer containing 1X ZOOM® 2D protein solubilizer, 2 M DTT, ZOOM® carrier ampholytes (Invitrogen, Carlsbad, USA) and bromophenol blue.

First dimension—isoelectric focusing (IEF): 155 μl of the protein in sample rehydration buffer was used to rehydrate each ZOOM® strip pH 3-10NL overnight using the ZOOM® IPGRunner™ cassettes (Invitrogen, Carlsbad, USA). After rehydration, isoelectric focusing was performed using the ZOOM® IPGRunner™ and the XCell SureLock™ mini-cell. Electrofocusing conditions were 200 V for 20 minutes, 450 V for 15 minutes, 750 V for 15 minutes and finally 2,000 V for 4 hours.

Second dimension—SDS-PAGE: IEF strips were equilibrated with NuPAGE® LDS sample buffer and NuPAGE® sample reducing agent and then alkylated with alkylation solution containing NuPAGE® LDS sample buffer and iodoacetamide (Biorad, Hercules, USA). The equilibrated strips were then applied to second dimension gels (Nu NuPAGE® Novex® 4–12% Bis-Tris ZOOM® gels) for SDS-PAGE at 200 V for 40–50 minutes using the NuPAGE® XCell SureLock™ mini cell system (Invitrogen, Carlsbad, CA).

Protein Transfer and immunodetection: The iBlot® dry blotting system was used for protein electroblotting of proteins to nitrocellulose membranes at 20 V for 7.5 minutes. Membranes were incubated with pre-immune or immune Tasmanian devil serum [2] at a dilution of 1:2000 in TBS buffer containing 0.1% tween 20 and 5% skim milk (TBSTM). After four washes with TBST, the membranes were incubated with a secondary polyclonal rabbit anti-devil immunoglobulin [29] at a dilution of 1:7500 in TBSTM. The membrane was finally incubated with a donkey horseradish peroxidase (HRP)-conjugated anti-rabbit IgG tertiary antibody (Amersham Biosciences, Piscataway, NJ) at a dilution of 1:5000 in TBSTM. A negative control omitting the primary antibody (devil’s serum) was also run. Chemiluminescent detection was performed with Immobilon™ Western HRP substrate (Merck Millipore, Billerica, USA) for 5 minutes and imaged using a Chemi-Smart 5000 digital camera (Vilber Lourmat, EEC).

#### Mass spectrometry analysis and selection of proteins

In-gel digestion: 2DE gels were stained with GelCode™ Blue Safe Protein Stain (Thermo Scientific, Rockford, USA) according to the manufacturer’s instructions. Matched immunospots were identified in the gel, excised with a scalpel and transferred to microcentrifuge tubes. Destaining of the spots and in-gel protein digestion with trypsin was performed using the In-Gel Tryptic Digestion Kit according to manufacturer’s guidelines (Thermo Scientific, Rockford, USA).

Mass spectrometry and protein identification: Peptide samples were analysed using an LTQ-Orbitrap tandem mass spectrometer and Surveyor MS Pump Plus (ThermoFisher Scientific, San Jose, CA), using the methodology for peptide separation and detection as previously described [30]. Briefly, peptides were separated using a 10 cm PicoFrit analytical nanoHPLC column packed with 5 μm C18 media (New Objective) over a 90 minute step gradient from 100% buffer A (5% acetonitrile in water containing 0.1% formic acid) to 100% buffer B (90% acetonitrile in water containing 0.1% formic acid). The LTQ-Orbitrap was controlled using Xcalibur 2.1 software in data-dependent mode and MS/MS spectra were acquired using a Top8 method with 30-second dynamic exclusion of fragmented peptides.

RAW mass spectrometry files were processed using MaxQuant version 1.5.1.2 (http://maxquant.org/) without using the ‘match between runs’ function and MS/MS spectra were searched against the *Sarcophilus harrisii* UniProt reference proteome (UP000007648; updated on 11/02/2016) using the Andromeda search engine. Settings for protein identification by LTQ-Orbitrap MS/MS allowed for a maximum of two missed trypsin cleavages, variable methionine oxidation, fixed carbamidomethylation and mass error tolerances of 20 ppm then 4.5 ppm for initial and main peptide searches, respectively, and 0.5 Da tolerance for fragment ions. A 1% false discovery rate was used for peptide-spectrum matches and protein identification. Where multiple proteins were reported (e.g. tubulin isoforms) peptides were assigned to satisfy the principal of parsimony.

## Results

### 1. Patterns of HSP expression in Tasmanian devil skin, primary tumours and DFTD cell lines

**Fig 1:**
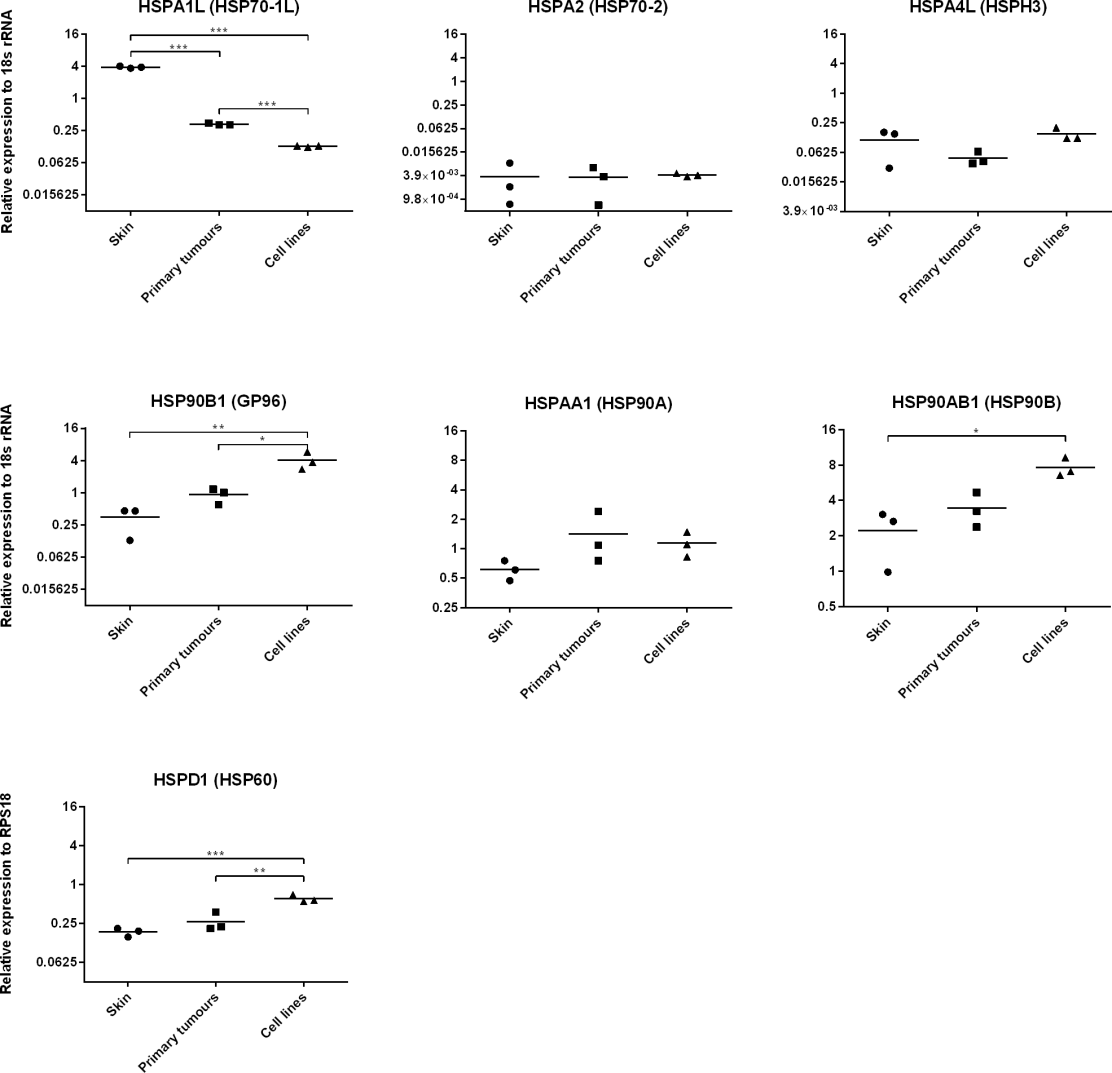
Expression of selected HSPs genes at basal level in normal Tasmanian devil skin, primary DFTD tumours and DFTD cell lines. Expression of the HSPS genes *HSPA1L*, *HSPA2*, *HSPA4L*, *HSP90B1*, *HSPAA1*, *HSP90AB1*, *HSPD1*, *and HSPB1* was analysed using qRT-PCR. Expression was measured relative to the control gene *RPS18*. Results are the mean and standard error of three biological replicates. Significance is defined as * *p*<0.05, ** *p*<0.01 and *** *p*<0.001. No-cDNA controls and no-RT controls were also included and did not show any amplification of product. Expression of *HSPB1* was not detected at basal level therefore, the figure is not shown. Results are representative of two experiments.

To study the expression of HSPs in the Tasmanian devil, we selected a range of genes belonging to the major HSP families that are annotated in the Tasmanian devil reference genome (Devil7.0 assembly GCA_000189315.1) (Table 1). We use quantitative reverse transcriptase polymerase chain reaction (qRT-PCR) to determine the expression of these genes in normal tissue (skin), primary DFTD tumours and three DFTD cell lines. We found that most of the HSPs tested were expressed at various levels across all biological replicates (Fig 1). We were unable to detect basal expression of *HSPB1* (HSP27) and have not included this gene in the figure. Primary tumours and skin showed similar base line levels of HSP expression. In comparison, *HSP90B1* (GP96), *HSP90AB1* and *HSPD1* (HSP60) were expressed at significantly higher levels in tumour cell lines than skin. *HSP90B1* and *HSPD1* were also expressed at higher levels in cell lines than primary tumours. In contrast, HSPA1L (HSP70-1L) was expressed at higher levels in skin than primary tumours and cell lines. These results confirm that a range of HSP genes are active in the Tasmanian devil and DFTD.

**Table 1:** Selected study heat shock protein (HSP) genes annotated in the Tasmanian devil reference genome.

Gene symbol*	Name*	Other names	Description**
**HSP70 superfamily**			
*HSPA1L*	Heat shock protein family A (Hsp70) member 1 like	HSP70-1L	This protein stabilizes existing proteins against aggregation and mediates the folding of newly translated proteins in the cytosol and in organelles.
*HSPA2*	Heat shock protein family A (Hsp70) member 2	HSP70-2, HSP70-3	This protein plays a pivotal role in the protein quality control system, ensuring the correct folding of proteins, the re-folding of misfolded proteins and controlling the targeting of proteins for subsequent degradation.
*HSPA4L*	Heat shock protein family A (Hsp70) member 4 like.	HSPH3, HSP70A4L	Heat shock inducible protein and may act as a chaperone. This protein can protect the heat-shocked cell against the harmful effects of aggregated proteins.
**HSPB family (small heat shock proteins)**			
HSPB1	Heat shock protein family B (small) member 1	HSP27, Hsp25	This protein is induced by environmental stress and developmental changes. The protein is involved in stress resistance and actin organization.
**HSP90 family**			
*HSP90AA1*	Heat shock protein 90 alpha family class A member 1	HSP90A, HSPC1, HSPCA	This protein is an inducible molecular chaperone. The protein aids in the proper folding of specific target proteins by use of an ATPase activity that is modulated by co-chaperones.
*HSP90AB1*	Heat shock protein 90 alpha family class B member 1	HSP90B, HSPC3	Molecular chaperone that promotes the maturation, structural maintenance and proper regulation of specific target proteins. It is the constitutive form of the cytosolic 90 kDa heat-shock protein. Undergoes a functional cycle that is linked to its ATPase activity.
*HSP90B1*	Heat shock protein 90 beta family member 1	GP96, Grp94, HSPC4	Member of a family of adenosine triphosphate (ATP)-metabolizing molecular chaperones with roles in stabilizing and folding other proteins. The protein is localized to melanosomes and the endoplasmic reticulum. Expression of this protein is associated with a variety of pathogenic states, including tumour formation.
**Chaperonins family**			
*HSPD1*	Heat shock protein family D (Hsp60) member 1	HSP60	Mitochondrial protein that may function as a signalling molecule of the innate immune system. It is essential for the folding and assembly of newly imported proteins in the mitochondria.

*Nomenclature according to the HUGO Gene Nomenclature Committee (HGNC).

** Description provided by NCBI and UniProt.

**Fig 2:**
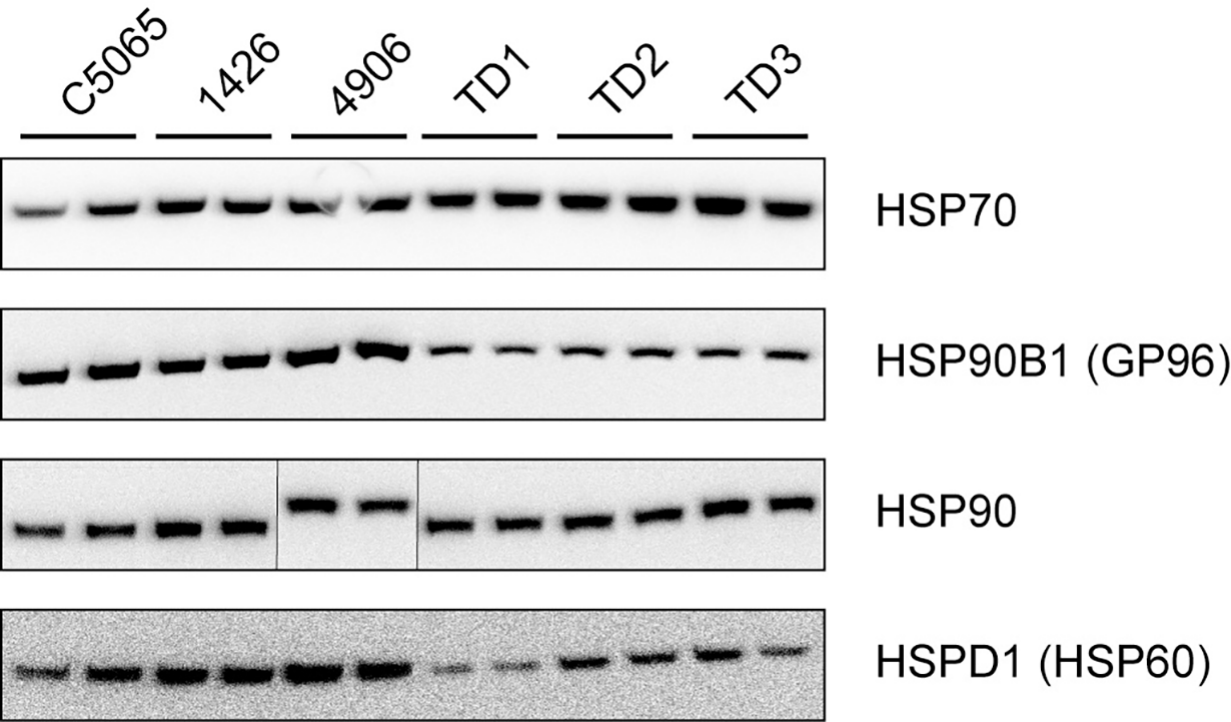
Expression of selected HSP proteins at basal level in DFTD cell lines and DFTD primary tumours. HSPs expression was detected by western blot using total protein isolated from three DFTD cell lines (C5065, 1426 and 4946) and three primary DFTD tumour biopsies (TD1, TD2 and TD3). The anti-HSP70 antibody detects different members of the HSP70 family; the anti GRP94 antibody detects the HSP90B1, the anti-HSP90 antibody detects various members of the HSP90 family and the anti-HSP60 detects the HSPD1. Bands represent two technical replicates for each biological sample. Bands from the same blot in the HSP90 panel were reorganised (vertical black lines) to have a consistent order in the figure. Original images of the blots are presented in S4 Fig. Results are representative of two experiments.

We then investigated the expression of HSPs at the protein level in three DFTD cell lines and three primary DFTD tumours using western blotting. We used commercially available antibodies that showed species cross reactivity with the Tasmanian devil. We used an anti-HSP70 antibody that detects different members of the HSP70 family; an anti-HSP90 antibody that detects members of the HSP90 alpha and beta families; an anti-HSP60 that detects HPSPD1, and an anti GRP94 that detects HSP90B1. We found HSP70, HSP90B1 (GP96), HSP90 and HSPD1 (HSP60) protein expression in both primary tumours and cell lines (Fig 2). The band intensity observed in the western blots suggested that levels of HSP90B1 and HSP60 protein expression are higher in cell lines than primary tumours, which correlates with the levels of expression detected at the mRNA level for these two genes.

Finally, we interrogated a DFTD cellular proteome that we recently acquired using shotgun proteomic analysis of the C5065 cell line [31] to confirm the presence of heat shock proteins in the tumour cells. This dataset comprised >1,000 proteins identified on the basis of two or more unique matching peptides and included 23 HSPs across four families: the HSP70 superfamily (6 proteins); the HSP90 family (3 proteins), the chaperonins (9 proteins) and the DNAJ/HSP40 family (5 proteins) (S3 Table).

### 2. HSP expression after heat shock treatment

**Fig 3:**
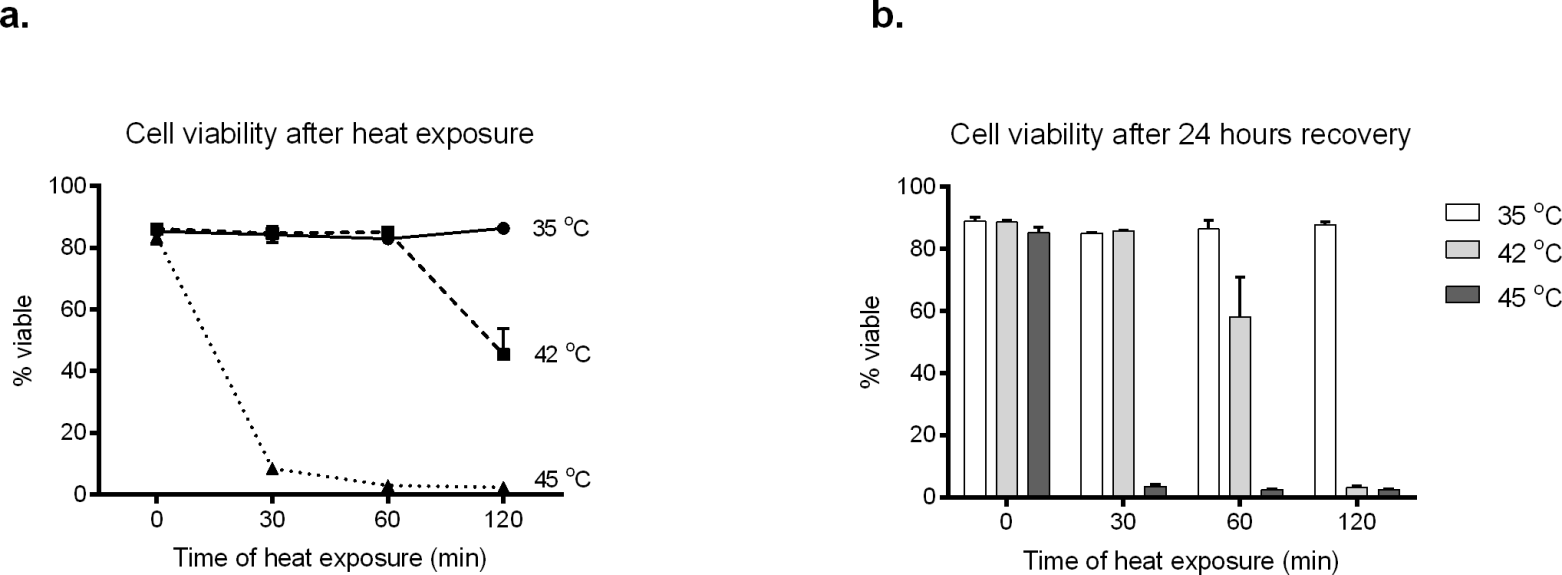
Studies of DFTD cell viability after heat shock treatment. **a**. DFTD cells growing in culture were exposed to heat shock (42 ^o^C and 45 ^o^C) or control temperature (35 ^o^C) in a water bath for 30, 60 or 120 min. Cell viability was analysed immediately after the treatment by flow cytometry. Each marker represents the mean and standard error of three biological replicates. **b.** DFTD cells in culture were treated as before but returned to normal incubation conditions (35 ^o^C) to allow recovery for 24 hours. Viability was then measured with flow cytometry. Bars represent the mean and standard error of three biological replicates. Experiments were repeated twice with reproducible results.

Several studies have indicated that changes in the expression of HSPs in response to heat shock treatment increases the immunogenicity of cancer cells (reviewed in [11]). Consequently we investigated whether the expression of HSPs in DFTD cells was upregulated by heat treatment. We initially determined the best conditions of heat shock treatment for the DFTD cell line C5065 (i.e. temperature, time of exposure and cell viability after the treatment) based on a protocol previously used in a human melanoma cell line [32]. As our DFTD cell cultures are maintained at 35 ^o^C, cells were treated with this temperature as a control. Cell viability, measured by flow cytometry, was not affected by heating the cultures at 42 ^o^C for 30 or 60 minutes, but was reduced to approximately 40% after 2 hours of treatment. In comparison, heat treatment at 45 ^o^C reduced the cell viability to less than 10% after the first 30 minutes of treatment (Fig 3A). We also examined cell viability after allowing the heat-treated cultures to recover under normal conditions (i.e. 35 ^o^C) for 24 hours (Fig 3B). Viability of the recovered cells that were treated at 42 ^o^C for 30 minutes was similar to the control. However, the viability of recovered cultures treated at 42 ^o^C for 60 and 120 minutes was reduced. Viability of cultures treated at 45 ^o^C after 24 hours recovery was lower than 10% of the control. From these experiments we selected 42 ^o^C for 30 minutes as the preferred treatment to study the effects of heat shock in the expression of HSPs in DFTD cells.

**Fig 4:**
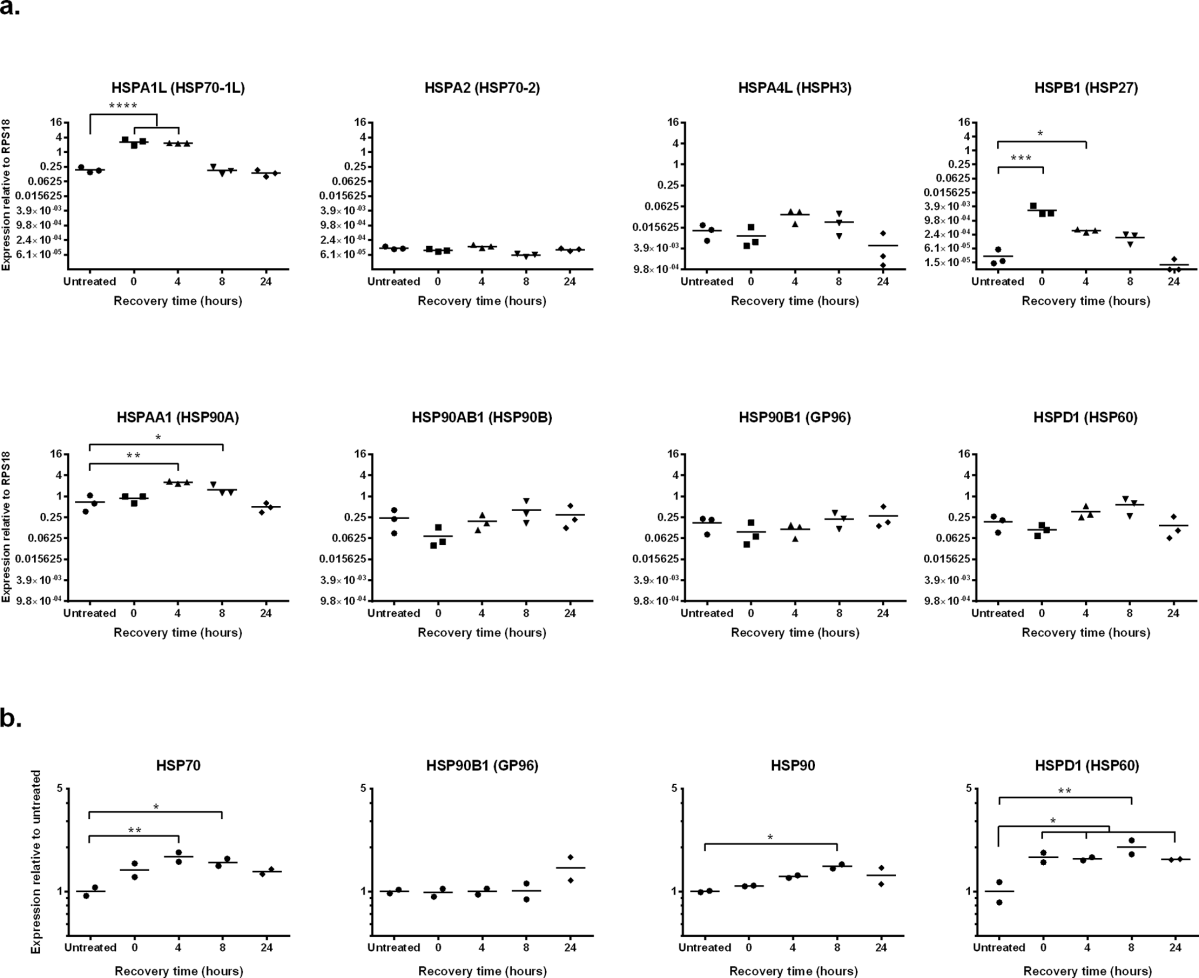
Expression of HSPs after heat shock treatment. Cultured DFTD cells were heat shocked at 42 ^o^C for 30 min in a water bath and then recovered in an incubator under normal cell culture conditions (35 ^o^C) for 4, 8, or 24 hours. **a**. HSP gene expression was analysed using qRT-PCR. Expression was measured relative to the control gene *RPS18*. Results are the mean and standard error of three biological replicates. Significance is defined as * *p*<0.05, ** *p*<0.01 and *** *p*<0.001. **b**. Protein expression of HSPs was analysed using western blot. Expression was measured relative to the untreated control. Results are the mean and standard error of two biological replicates. Statistical significance is defined as * *p*<0.05, ** *p*<0.01 and *** *p*<0.001. Results are representative of two experiments.

DFTD cells were heat treated as indicated above and HSP expression was determined by qRT-PCR after different lengths of recovery (0, 4, 8 and 24 hours). Expression of *HSPA1L* (HSP70-1L), *HSPB1* (HSP27) and *HSPAA1* (HSP90A) was significantly increased after heat treatment (Fig 4A). While upregulation of *HSPA1L* and *HSPB1* was detected immediately after the treatment, significant upregulation of *HSP9AA1* was only detected 4 hours after the treatment. Upregulation of the three genes was transient and expression of the HSPs returned to similar levels as the untreated control after 24 hours of recovery at 35 ^o^C. These results confirm the presence of at least three heat-inducible genes belonging to the HSP70 and HSP90 superfamilies (*HSPA1L* and *HSPAA1*, respectively) and the small heat shock protein family (*HSPB1*) in Tasmanian devils.

We also used western blot to study changes to the levels of protein expression in DFTD cells after heat treatment. Significant upregulation of HSP70 was observed at 4 and 8 hours after treatment (Fig 4B). HSPD1 (HSP60) was significantly upregulated immediately after heat treatment and was maintained at a heightened level for the 24 hours of recovery time. The level of protein expression of HSP90 increased after the treatment and was significantly higher than the control after 8 hours of recovery. Increased HSP90B1 protein expression was observed only at 24 and was not statistically significant.

### 3. HSP expression after radiation exposure

**Fig 5:**
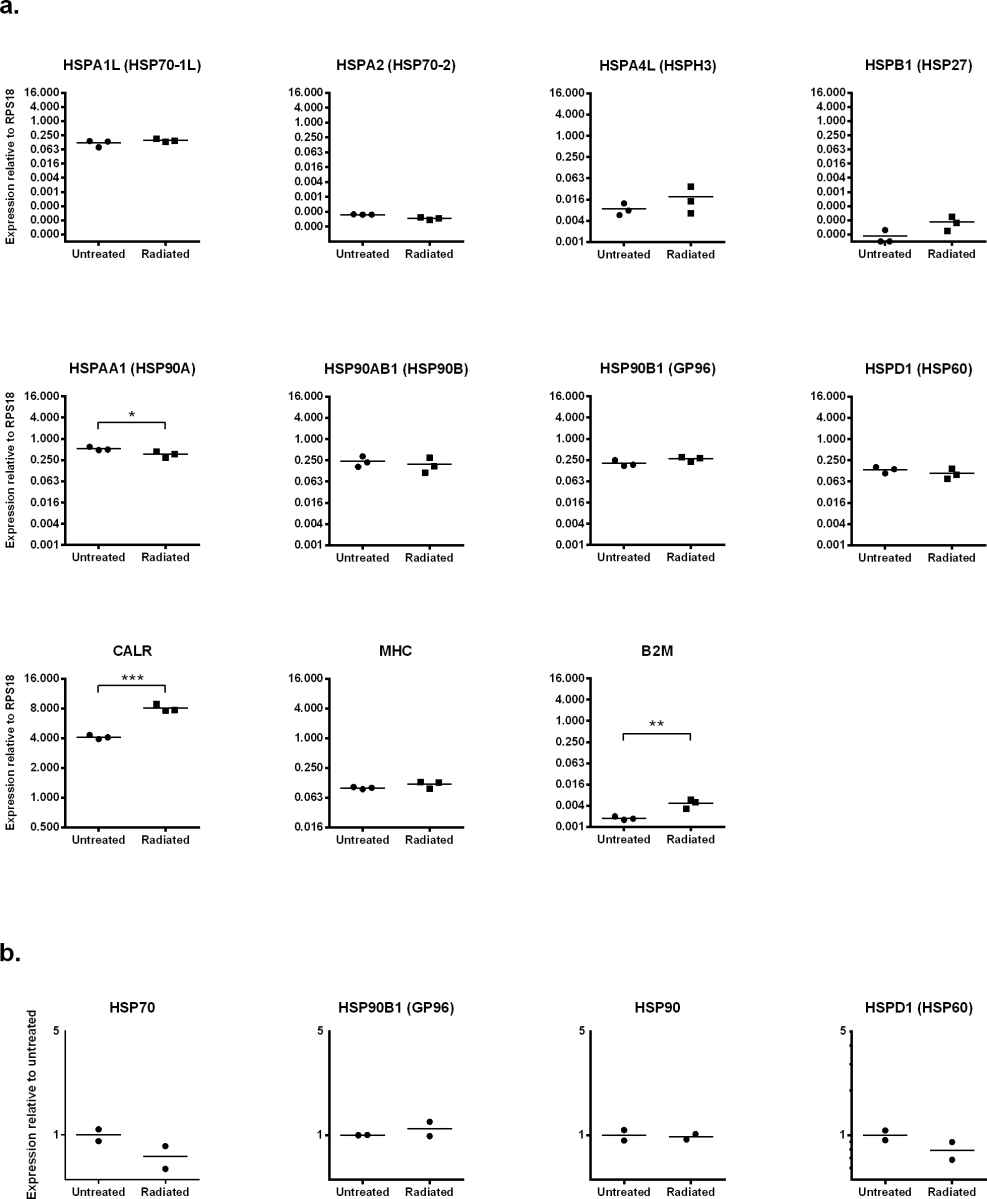
HSP expression after radiation. Cultured DFTD cells were exposed to gamma radiation (20 Gy) and then recovered for 24 hours in an incubator under normal cell culture conditions. **a.** HSP gene expression was analysed using qRT-PCR. Expression was measured relative to the control gene *RPS18*. Results are the mean and standard error of three biological replicates. Significance is defined as * *p*<0.05, ** *p*<0.01 and *** *p*<0.001. **b**. Protein expression of HSPs was analysed using western blot. Expression was measured relative to the untreated control. Results are the mean and standard error of two biological replicates. The results from one single experiment are shown. Statistical significance is defined as * *p*<0.05, ** *p*<0.01 and ***p<0.001.

Similar to other environmental stressors, radiation may increase the immunogenicity of cancer cells by upregulating the expression of HSPs [33–35] and by increasing antigen presentation [36, 37]. As we use radiation to inactivate DFTD cells for our vaccines [2, 3], we were interested in studying the effects of radiation on the tumour cells. We used qRT-PCR to investigate the expression of HSPs, calreticulin (*CALR*) (an “eat me signal” molecule expressed in the surface of cancer cells that promotes their removal by cells of the immune system), *B2M* and SAHAI-01, a classical MHC-I molecule of the Tasmanian devil [1, 38]. Of the eight HSPs analysed, significant changes were only observed in the expression of *HSPA1A* (HSP70-1a), which was upregulated, and *HSPAA1* (HSP90A), which was downregulated (Fig 5A). *CALR* and *B2M* were also significantly upregulated after radiation. At the protein level, we observed reduced expression of the HSP70 and HSPD1 (HSP60) after radiation, however, these changes were not statistically significant (Fig 5B).

### 4. HSPs as DFTD tumour associated antigens

Immunisation trials with non-viable DFTD cell lines by our research group have shown that Tasmanian devils can induce humoral responses against DFTD cells [2, 3]. We sought to identify the DFTD tumour antigens responsible for the antibody response and to investigate whether circulating antibodies against HSPs were present in the serum of the immunised devils. To achieve this, we used an immmunoproteomic approach based on separation of DFTD cellular protein extracts by 2-DE, protein transfer onto nitrocellulose membrane and detection using serum from immunised devils (primary antibody). Antigen-antibody reactions are then detected using a polyclonal anti devil immunoglobulin (secondary antibody) and a HRP-conjugated tertiary antibody. Corresponding protein spots of interest are excised from a coomassie blue-stained gel run in parallel and, after in-gel trypsin digestion, identified by mass spectrometry.

**Fig 6:**
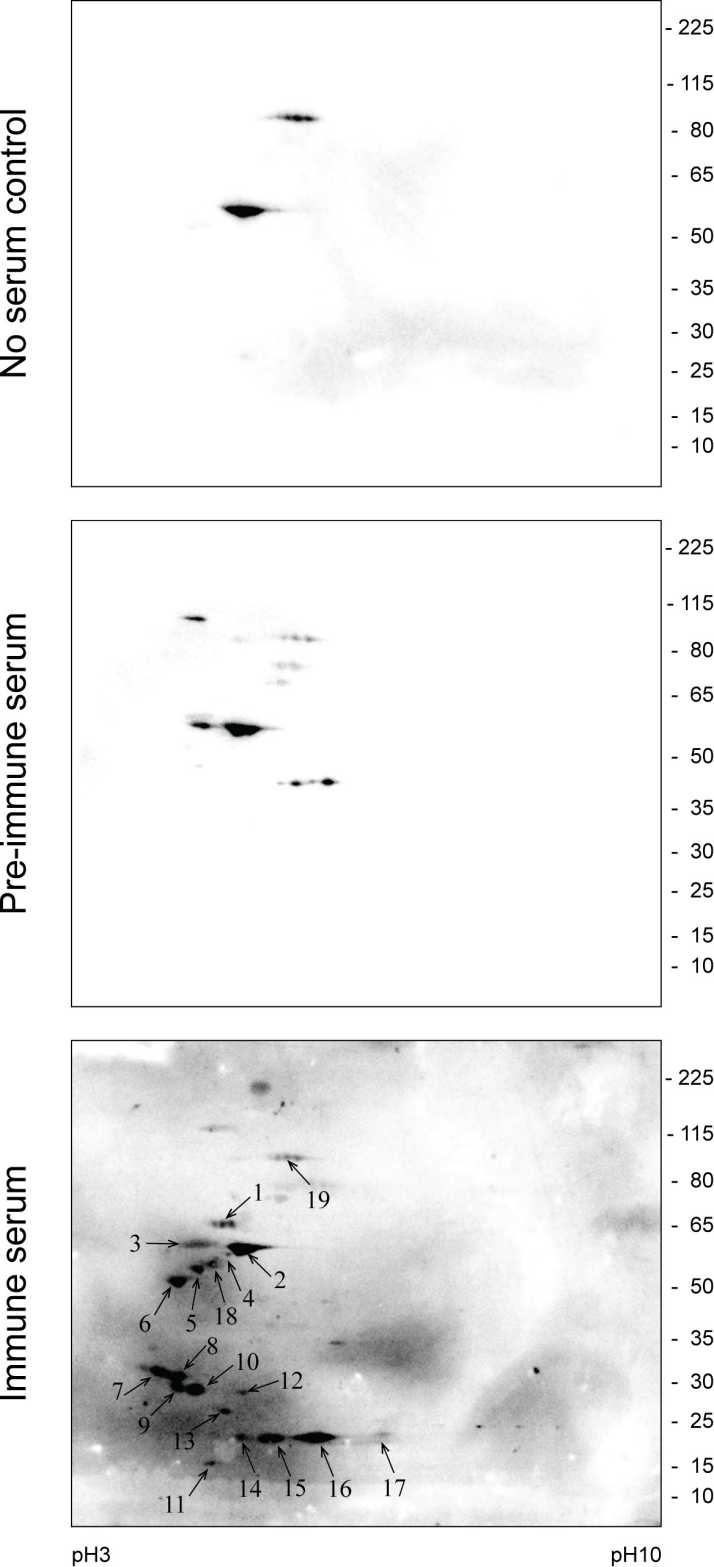
Screening of antibodies against DFTD tumour antigens using serum from an immunised Tasmanian devil. Total cell proteins from cultured DFTD cells were separated by two-dimensional gel-electrophoresis, transferred to nitrocellulose membrane and probed with pre-immune serum or post-immunisation serum. Negative control (no serum) is also shown. Arrows indicate spots detected by the immune serum that were selected for further identification by mass spectrometry (see Table 2). Experiments were repeated twice with reproducible results.

We repeated this analysis using the serum collected from two different immunised devils and the results presented here are representative. Total DFTD protein extracted from a cell line was separated by 2-DE in four gels run in parallel. Protein from three gels was transferred to nitrocellulose membranes. One membrane was used as a negative control (no serum control), the second membrane probed against pre-immune devil serum, and the third membrane probed with devil serum after immunisation (Fig 6). The western blots revealed a range of antigens only recognised by antibodies in the immune serum, this is, different from the background detected in the no-serum control (spots 2 and 19) or by the pre-immune serum (spot 3). All the 19 spots were identified and excised from the fourth 2-D gel and analysed by mass spectrometry, resulting in the identification of 27 different proteins. In some cases, several isoforms of a single protein were detected. In this case, all isoforms were collectively counted as a one protein (Table 2 and S4 Table). Literature searching using PubMed found that many of the proteins had been previously identified as tumour antigens or were associated with the immune response to cancer in other species. In particular, 11 proteins were found to have well-documented information for their relation to cancer, including the HSP60 proteins (S5 Table). These preliminary results indicate that it is feasible to use immunoproteomics to identify DFTD-associated antigens. Furthermore, this preliminary study shows that HSP60 proteins are antigenic and may contribute to the humoral response observed in our vaccination trials.

**Table 2:** DFTD tumour antigens recognised by serum of an immunised Tasmanian devil and identified by mass spectrometry.

Spot	Unique peptides	Amino acid coverage (%)	Mass (kDa)	Accession number	Gene symbol*	Protein name*
7,8,9	6	41.9	28	G3WCJ6	YWHAB	14-3-3 protein beta/alpha
7,8,9	7	39.9	29	G3WP04	YWHAE	14-3-3 protein epsilon
7,8,9	2	21.7	28	G3VE53	YWHAH	14-3-3 protein eta
7,8,9	5	34	28	G3VS43	YWHAQ	14-3-3 protein theta
7,8,9,10	11	54.7	28	G3WHE0	YWHAZ	14-3-3 protein zeta/delta
1	11	31.1	61	G3WZS7	HSPD1	60 kDa heat shock protein, mitochondrial
19	4	6.2	87	G3W8J9	ACO2	Aconitase hydratase, mitochondrial
10	2	12.3	24	G3W6S0	NUDT5	ADP-sugar pyrophosphatase
2,4,18	20	54.9	55	G3VIJ8	ATP5B	ATP synthase subunit beta, mitochondrial
12	4	14	37	G3W5Z0	CTSB	Cathepsin B
11	7	54.4	15	G3WZD1	CRABP1	Cellular retinoic acid-binding protein 1
19	3	6.1	80	G3WXK9	APPL1	DCC-interacting protein 13-alpha
2,4,5,6,18	2	6.6	53	G3WHB8	DES	Desmin
19	11	23.2	82	G3X0B8	GSN	Gelsolin
19	4	7.2	83	G3WYP8	HSP90AB1	Heat shock protein HSP 90-beta
1,2	5	16.5	51	G3WXL4	HNRNPK	Heterogeneous nuclear ribonucleoprotein K
14,15,16,17	3	27.8	13	G3VNV7	LOC100928127	Histone H2B
18	11	32.9	53	G3VGI4	PLIN3	Perilipin-3
17	3	21.4	22	G3VCM9	PRDX5	Peroxiredoxin-5, mitochondrial
7	5	34.9	28	G3WDY3	PCNA	Proliferating cell nuclear antigen
3	8	17.3	57	G3WBV2	P4HB	Protein disulfide-isomerase
10	6	41.2	23	G3WB53	ARHGDIA	Rho GDP dissociation inhibitor 1
16	4	23.3	17	G3VVY5	STMN1	Stathmin
17	3	23.1	15	G3VNM4	SOD1	Superoxide dismutase [Cu-Zn]
12	2	10.3	32	G3VNJ4	TMX1	Thioredoxin-related transmembrane protein 1
11	2	18.1	12	G3VX55	TXN	Thioredoxin
7	4	17.6	28	G3VU66	TPM4	Tropomyosin alpha-4 chain
2,4,16,18	2	33	50	G3WSM9	LOC100920562	Tubulin alpha chain
2,4,18	2	38.6	50	G3VPL9	TUBA1C	Tubulin alpha-1C chain
2,4,18	14	34.1	50	G3WFK2	TUBA8	Tubulin alpha-8 chain
4,18	5	42.3	50	G3W109	TUBB	Tubulin beta chain
4,18	2	35.5	50	G3WMM3	TUBB2A	Tubulin beta-2A chain
4,18	17	45.4	50	G3WJU5	TUBB4A	Tubulin beta-4A chain
4,18	6	40.6	50	G3VXT3	TUBB6	Tubulin beta-6 chain
13	2	12.7	25	G3WV76	UCHL1	Ubiquitin carboxyl-terminal hydrolase isozyme L1
18	6	22.2	45	G3WRR2	UBA5	Ubiquitin-like modifier-activating enzyme 5
1,2,3,4.5,6,18	25	77.2	53	G3WPL8	VIM	Vimentin

* Gene and protein names according to The Universal Protein Resource (UniProt).

## Discussion

In this study we present the first characterisation of heat shock proteins (HSPs) in the marsupial Tasmanian devil. We evaluated the suitability of these proteins as antigenic components of a vaccine against devil facial tumour disease (DFTD). Our results confirmed the constitutive expression of a wide range of HSPs in normal devil tissue, primary DFTD tumours and DFTD cell lines. We also confirmed the expression of stress-inducible HSPs in DFTD cancer cells. More importantly, antibodies present in the serum of immunised devils recognised a range of DFTD proteins including HSPs. These results support the feasibility of using HSPs preparations as components for the enhancement of a DFTD vaccine.

HSPs are considered one of the most phylogenetically conserved group of proteins [4]. In normal cells, HSPs play essential roles in the synthesis, transport and folding of proteins. In cancer cells, HSPs are also important regulators of cellular responses and functions during carcinogenesis [39]. Therefore, it was not surprising that basal HSP expression was detected both in the skin of the Tasmanian devil and in DFTD tumour cells. We confirmed expression of members of several HSP families described in the literature [40]. These included the HSP70 superfamily (HSPA and HSPH families), the HSPB family, the HSP90 family, the chaperonin family and the HSP40 (DNAJ) family.

Compared to normal devil skin, we found significantly higher expression of HSPD1 (HSP60) and two HSP90 proteins (HSP90B1 and HSP90AB1) in cultured DFTD cells. HSP60 and HSP90 are overexpressed in numerous human cancers and have been correlated with cancer progression and poor prognosis [41–49]. HSPs promote cancer development through the regulation of angiogenesis, cell proliferation, migration, invasion and metastases [50]. Additionally, cancer cells have higher metabolic requirements for chaperones than normal cells in order to resist stressors such as protein overload and hypoxia. DFTD is an aggressive cancer and metastases are frequent in affected animals. It is therefore possible that HSP60 and HSP90 exert similar cancer promoting functions in DFTD cells.

An important function of HSPs involves protecting cells from irreversible damage under stressful conditions that could lead to cell death [51]. In the current study, heat shock upregulated *HSPA1L* (HSP70-1L), *HSPB1* (HSP27), *HSPAA1* (HSP90A) and HSPD1 (HSP60), confirming that HSPs in DFTD cells are stress responsive. In eukaryotes, this rapid and classical stress response occurs via transcriptional induction of the heat shock genes via the heat shock transcription factor (HSF1), which binds highly conserved regulatory sequences (heat shock elements, HSEs) located within heat shock gene promoters [52]. Interestingly, our study showed that cancer cells of the marsupial Tasmanian devil upregulate *HSPA1L* after heat shock. In mammals, the HSP70 family genes belonging to the *HSPA1* cluster include three genes. Two of these genes, *HSP1A* and *HSPA1B*, contain HSEs in their regulatory regions and their expression is strongly increased in most tissues after heat shock and other stressors. The third gene, *HSP1AL*, is localized in close vicinity to *HSPA1A* but not contain HSEs in the promoter; in humans this gene is not inducible [53]. Comparative studies of the regulation of the heat shock promoters in insects and mammals show that the heat shock regulation system is not universal for distant species, and the variability in the evolution of individual heat shock genes may depend on environmental conditions. For example, studies in camels, which are adapted to live in arid desert areas, show that tissues of this species constitutively express all three members of the HSPA1 cluster under normal physiological conditions. Moreover, the study showed that heat shock also increases the expression levels of the three members of the cluster. Although the regulatory mechanisms responsible for the increased level of the expression of the constitutive HSPA1L (that lacks heat shock elements) are unknown, it was suggested that the genes in the cluster HSPA1 may interact functionally with each other [54]. If this is the case for the regulation of heat shock expression in the Tasmanian devil and other marsupials, further investigation is required.

I addition to the regulatory mechanisms present at the transcriptional level, the expression of HSP is also highly controlled at the post-transcriptional level [55–57]. Our results suggest the existence of this machinery in DFTD cells. We observed elevated protein expression of HSP60 immediately after heat treatment but we were unable to detect variation at the mRNA level. It has been suggested that eukaryotic translation initiation factors (eIF) and other mechanisms influence the translation of HSP after heat shock. Studies in HeLa cells deficient in eIF-4E and eIF-4γ, detected newly synthetised HSP70 and HSP90 without increase in respective mRNAs levels. The study suggested that HSPs are translated by cap-independent pathways [56]. In contrast to our observation, a study using human prostate carcinoma cell lines showed that despite an increase in HSP60 mRNA after heat shock, no increase in HSP60 protein was detected [58]. The reason for this discrepancy is not known. It may indicate different regulatory mechanisms at the cellular level or perhaps at the species level which defines topics for future research.

Radiation is another environmental pressure that triggers a cellular stress response with implications for cancer progression. As a cancer therapy, radiation influences a number of biological and immunological functions in cancer cells leading to contrasting outcomes. Radiation induces the expression of HSPs that protect cancer cells by inhibiting radiation-induced apoptosis [59]. On the other hand, radiation induces overexpression of HSP70, HSP90 and calreticulin (*CALR*), which promotes anti-tumor immunity [34]. Furthermore, radiation may increase antigen presentation by upregulating MHC-I expression [37]. Our current whole-cell vaccination approach uses high doses of radiation (two daily doses of 40 Gy each) as a safety measure to completely inactivate the DFTD cells prior immunisation of Tasmanian devils [3]. In this study, we used a lower radiation level of 20 Gy to study its effect on HSP expression in DFTD cells. We were not able to detect changes in HSP expression at the mRNA or protein level after the radiation treatment. These data is in line with others studies indicating that radiation is not a powerful inducer of HSP expression [58]. On the other hand, radiation upregulated the gene expression of *B2M* and *CALR* suggesting that this treatment may be used as a mechanism to increase the immunogenicity of DFTD cells. Several studies have showed that the translocation of calreticulin to the plasma membrane induced by radiation is critical for the recognition of dying tumour cells by dendritic cells [36, 60]. Additionally, surface expression of calreticulin is also correlated with an increased antitumor immune response by natural killer cells and effector memory CD4^+^ and CD8^+^ T cells [61]. The use of lower doses of radiation in combination with mechanical inactivation of the cells (e.g. freezing/thawing) may be a feasible strategy to increase the immunogenicity of DFTD cells and the efficacy of our current DFTD vaccine.

Our study provides preliminary evidence that HSPs in DFTD are immunogenic. In particular, we identified HSP60 as tumour antigen recognised by the serum from a devil immunised with DFTD tumour cells. These findings show that immunoproteomics is a viable technique for characterisation of specific DFTD tumour antigens. Although the results are preliminary, the antigens identified using this technique may provide clues about tumourigenic processes occurring in this unique cancer and could be useful for the development of an anti-DFTD vaccine. Some of the identified antigens relate to pathways considered “hallmarks of cancer cells” [62]. Biological processes include: *cell cycle progression* (14-3-3 proteins), *signal transduction* (HSP90 and HSP60), *cellular bioenergy* (aconitate hydratase and ATP synthase), *proliferation* (14-3-3 proteins, cathepsin B, cellular retinoic acid, gelsolin and proliferating cell nuclear antigen), *migration* (gelsolin and cathepsin B) and *apoptosis* (14-3-3 proteins, HSP60 and DCC-interaction protein 13-alpha). Future studies are required to confirm the role and possible therapeutic applications of these proteins in DFTD.

Taking together, our results support the feasibility of using HSP preparations derived from DFTD cells as an alternative, or to complement, the current whole-tumour cell formulation. Several approaches to separate HSPs from tumour cells for use in cancer vaccines have already been developed and tested. The efficacy of autologous HSP preparation as an immunotherapy was initially demonstrated by Tamura *et al*., in 1997 [63]. Their study showed that tumour-derived GP96 (HSP90B1) and HSP70 preparations were able to eradicate established tumours in animal models. Moreover, GP96 preparations derived from metastatic lesions were also effective in conferring long term protection against the primary tumour. The anti-tumour efficacy is derived from the ability of the HSP to chaperone the entire antigenic repertoire of the cells from which they are isolated. Antigen presenting cells can take up HSP-peptide complexes and present the antigenic peptides to CD8^+^ and CD4^+^ T cells [10]. Following these studies, various clinical trials with cancer patients have been initiated (reviewed in [8, 11]). The first autologous cancer-derived vaccine based on GP96 is known as Vitespen® and has shown promising results in patients with melanoma and kidney cancer (reviewed in [64]). More recently, HSP peptide complex–96 (HSPPC-96) has been tested in a phase II study of patients with recurrent glioblastoma multiforme (GBM). The study indicated that the vaccine is safe and its efficacy encouraging [65]. Similar studies using HSP70-peptide complexes have been tested in patients with leukemia and other cancers [8, 66].

DFTD cells lack components of the antigen presenting machinery [1], therefore our current vaccine incorporates DFTD cells treated with cytokines to restore surface MHC-I expression. A downside to this approach involves the concurrent upregulation of the immune-inhibitory molecule PD-L1 with MHC-I [67]. In this sense, HSP preparations are advantageous as they may carry a wide repertoire of antigenic peptides derived from the tumour cells independently of MHC-I [9]. A constraint of using HSP-peptide complexes for human therapy is the limited availability of tumour-derived tissue from cancer patients. Conversely, DFTD is a clonal cancer, therefore vaccination can include a common batch of HSP preparations that are purified from readily available cell lines or tumour biopsies. Further research in the development of HSP-vaccine against DFTD in Tasmanian devils is warranted.

The main objective of this study was to evaluate the potential of using HSPs as an antigenic substitute to whole tumour cell extracts in DFTD vaccines. Here we have shown that tissues and cancer cells from the Tasmanian devil express constitutive and stress-inducible HSPs. More importantly, our study suggests that HSPs derived from DFTD cancer cells are immunogenic and are recognised by the devil’s immune system. Taken together, our findings pave the way for the future development of HSP-based vaccine against DFTD.

## Supporting information

S1 Fig Titration of antibodies. Optimal amount of protein to load in the gels was determined for each antibody. Each sample was run in duplicate (four top panels). A second gel was run in parallel to determine total protein using SYPRO® Ruby Protein Gel Stain (bottom panels). ImageQuant TL 8.1 software was used for densitometry analysis of the blots and gels.
(TIF)

S2 Fig Band analysis for protein quantitation. (TIF)

S3 Fig Gating strategy used for discriminating and quantifying viable and non-viable cells. (TIF)

S4 Fig Original blots. (TIF)

S1 Table Primers used for qRT-PCR. (PDF)

S2 Table Antibodies used in the study. (PDF)

S3 Table HSPs identified in the proteome of a DFTD cell line. (PDF)

S4 Table MaxQuant output txt files. (XLSX)

S5 Table Biological relevance of selected cognate DFTD antigens identified by mass spectrometry analyses. (PDF)
