# Supplementary figures and images for: Heat shock proteins expressed in the marsupial Tasmanian devil are potential antigenic candidates in a vaccine against devil facial tumour disease
Source: PLoS One. 2018 Apr 27;13(4):e0196469. doi: 10.1371/journal.pone.0196469 (PMC5922574; doi:10.1371/journal.pone.0196469)

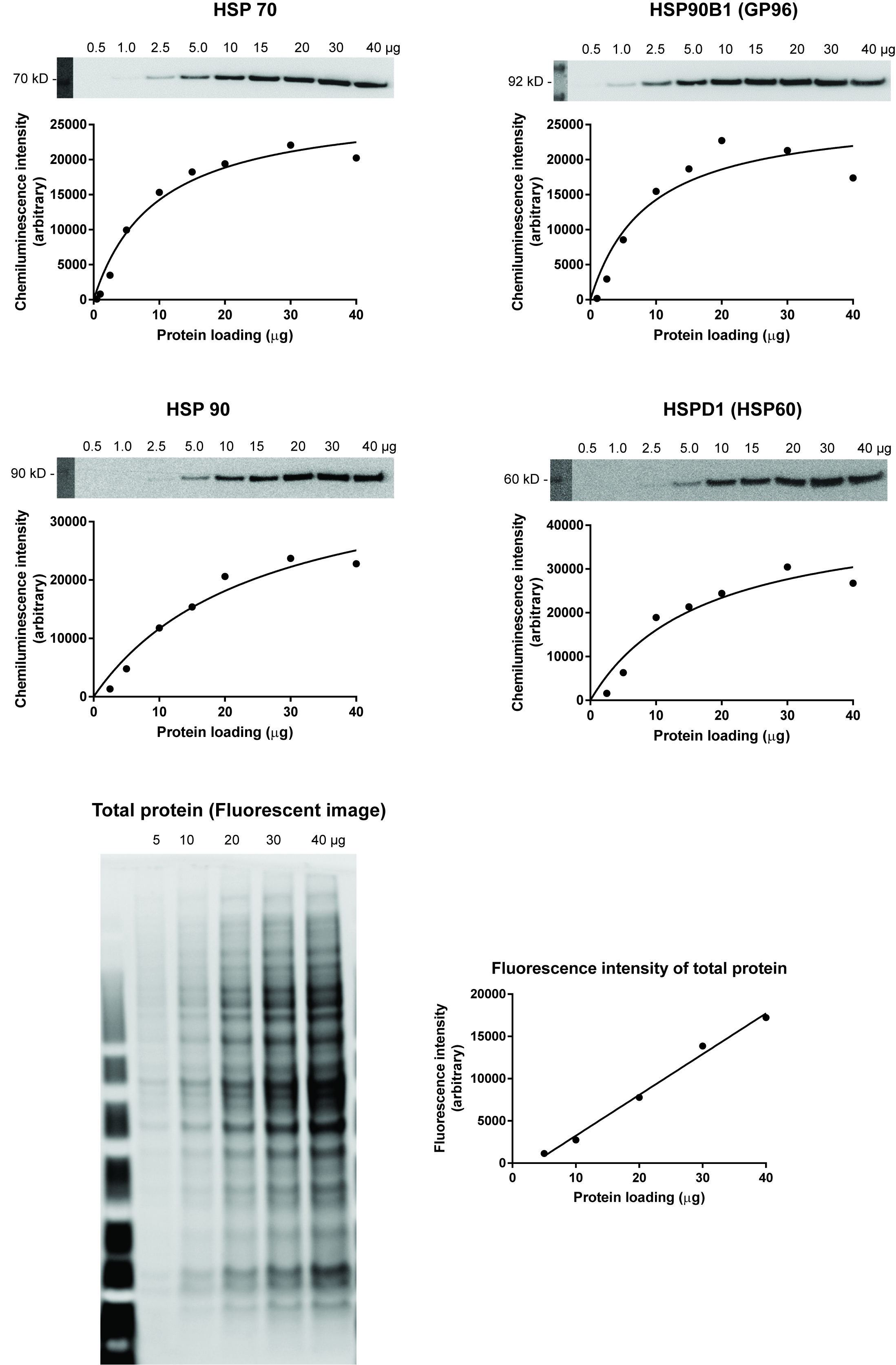

Supplement: S1 Fig — Optimal amount of protein to load in the gels was determined for each antibody. Each sample was run in duplicate (four top panels). A second gel was run in parallel to determine total protein using SYPRO® Ruby Protein Gel Stain (bottom panels). ImageQuant TL 8.1 software was used for densitometry analysis of the blots and gels. (TIF) [file pone.0196469.s001.tif]

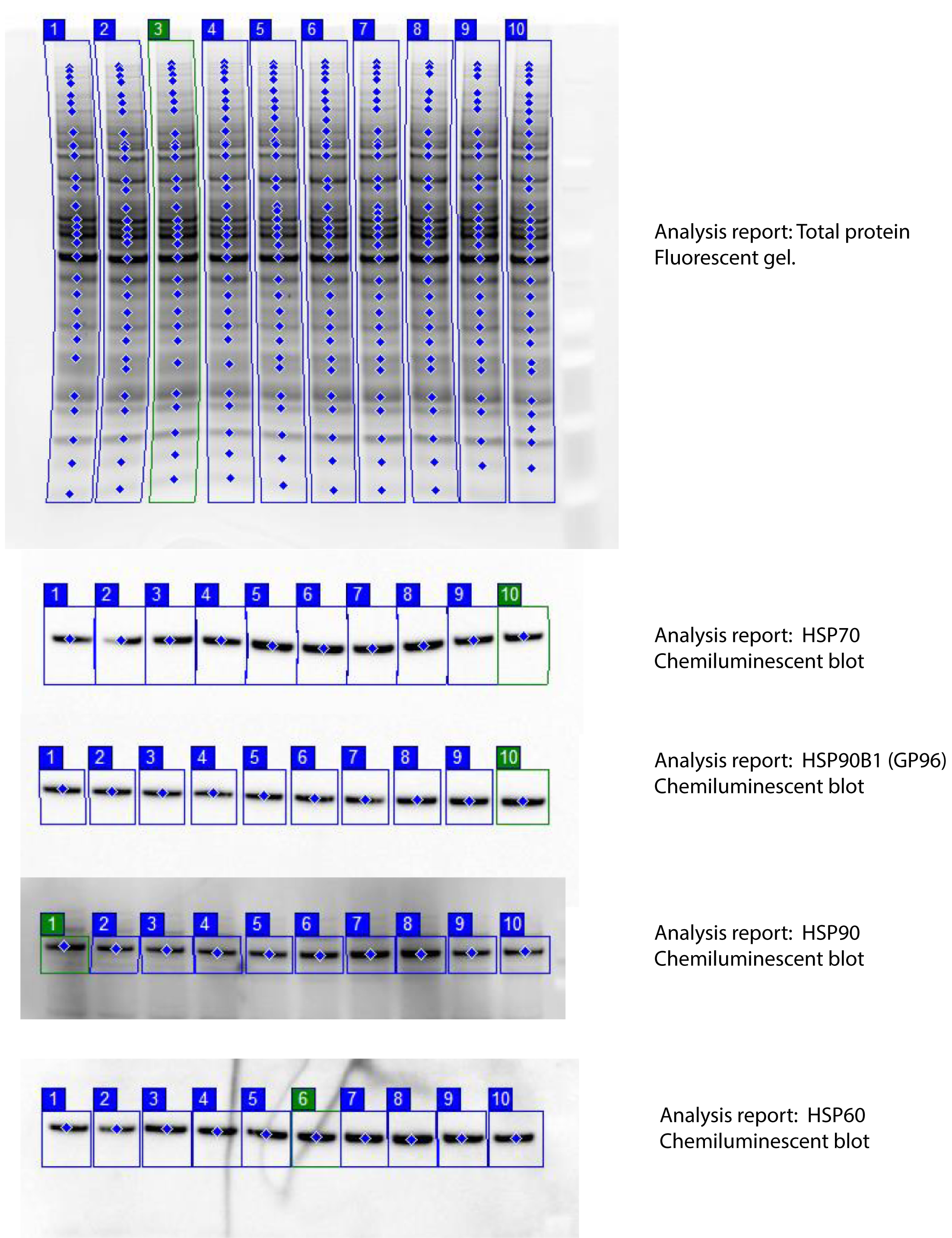

Supplement: S2 Fig — (TIF) [file pone.0196469.s002.tif]

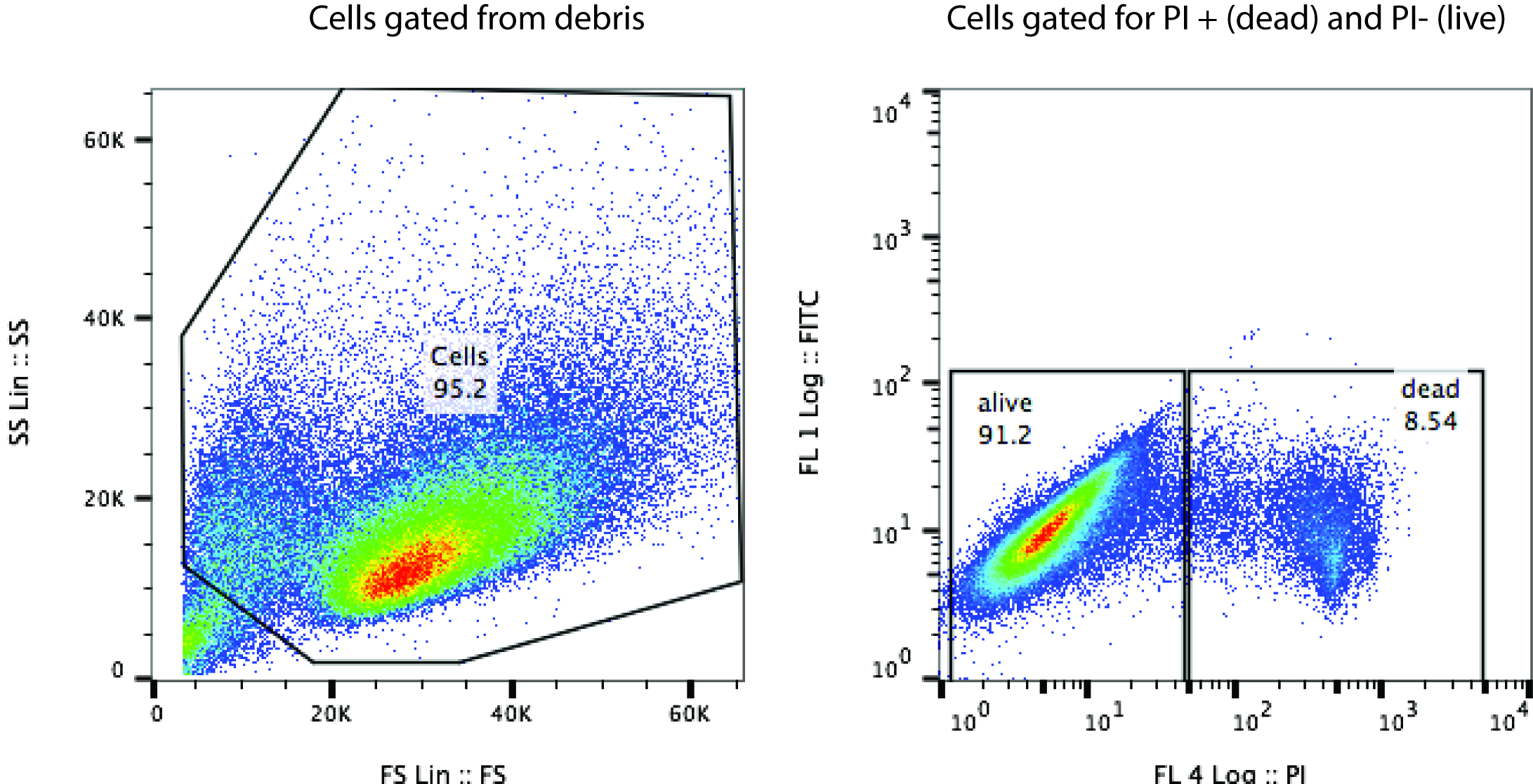

Supplement: S3 Fig — (TIF) [file pone.0196469.s003.tif]

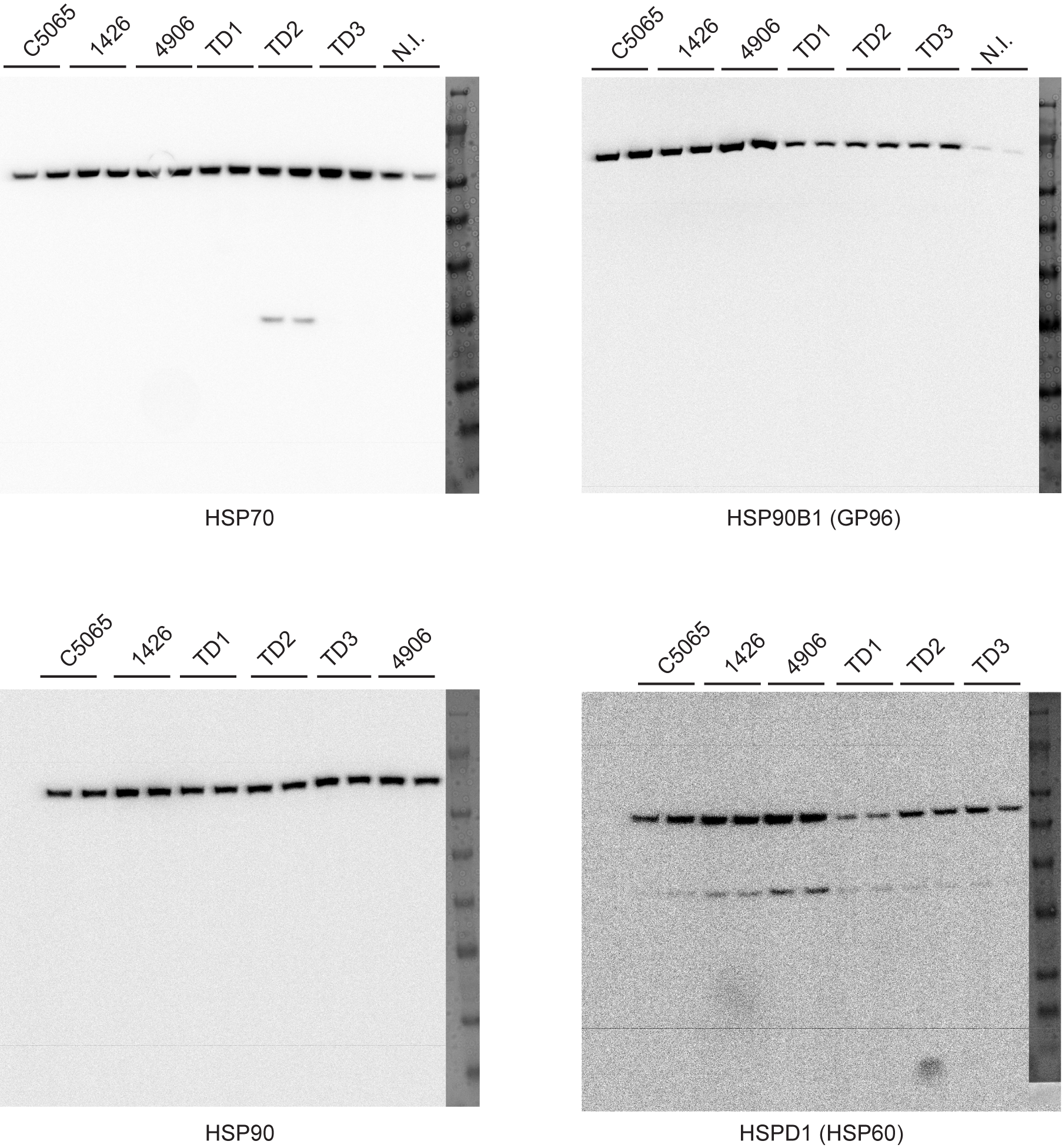

Supplement: S4 Fig — (TIF) [file pone.0196469.s004.tif]
