# Supplementary material for: Heat shock proteins expressed in the marsupial Tasmanian devil are potential antigenic candidates in a vaccine against devil facial tumour disease
Source: PLoS One. 2018 Apr 27;13(4):e0196469. doi: 10.1371/journal.pone.0196469 (PMC5922574; doi:10.1371/journal.pone.0196469)
Supplement: S1 Table — (PDF) [file pone.0196469.s005.pdf]

S1 Table. Primers used for qRT-PCR

Gene	Forward (F) and Reverse (R) Primers	Estimated PCR product size (bp)	Sequence accession number (NCBI and Ensembl)
RPS18	F AGCGGCTGAAGAAGATACGG<br>R TTGGACACACCCACAGTACG	115	XM_003768950.3
HSPA1L<br>(HSP70-1L)	F AGAGACCTGAACAAGAGTATCAACC<br>R AGGTCCTGCACTTTCTCTGATTTAT	80	XM_003768911.3
HSPA2<br>(HSP70-2)	F GTATTGAAACCGCAGGGGA<br>R GACACTGCTCTGGTTGTCG	111	XM_003756345.2
HSPA4L<br>(HSPH3)	F ACATGAAGAGAGACCGAAAGC<br>R CAGGCTTACTTTCAGGCCCA	380	XM_023505624.1
HSPB1<br>(HSPB1-201,<br>HSP27)	F ACATTTGCTCGGTCACTCCC<br>R AAGCCGTGCTCATCTTGTCT	121	ENSSHAG000000125<br>27/ENSSHAT000000<br>14799.1
HSPAA1<br>(HSP90A)	F TTGGTTACTTCCCCGTGCTG<br>R GCCTTTTGCCGTAGGGTTTC	176	XM_003756541.3
HSP90AB1<br>(HSP90B)	F TATCCGAGGTGTGGTGGACT<br>R GCTTCCGTACTCGTTCCACA	389	XM_012549073.2
HSP90B1<br>(GP96)	F TTGTTCCCACTTCTGCTCCC<br>R AGCCGAGTACGGTTGGAATG	355	XM_003772592.2
HSPD1<br>(HSP60)	F AGGCAAGGGTGAAAAATCCCA<br>R AAGCAATGCACAACCACCAC	259	XM_012544896.2
CALR	F GATGGGGAATGGGAACCACC<br>R CAGGGGTGTACTCAGGGTTG	133	XM_003760668.3
MHC Class 1<br>antigen<br>(SAHAI-01)	F CAGATTTCCCGAGTGGAC<br>R GTCGTAGGCGAACTGAAG	144	NM_001280855.1
B2M	F TGTGCATCCTTCCCTACCTGGAGG<br>R CATTGTTGAAAGACAGATCGGACCGC	201	XM_003756064.3

RPS18, ribosomal protein S18.

Supplementary Table 2. Antibodies used in the study

Antibody	Supplier	Antibody Registry number	Dilution
Polyclonal goat anti HSP60	Abcam (Ab82520)	AB_1658326	1:500
Monoclonal mouse anti HSP70	Abcam (Ab2787)	AB_303300	1:1000
Polyclonal rabbit anti HSP90	Abcam (Ab13495)	AB_1269122	1:1000
Monoclonal rat anti Grp94	Abcam (Ab90458)	AB_2295611	1:1000
Polyclonal rabbit anti β-actin	Abcam (Ab8227)	AB_2305186	1:1000
Polyclonal goat anti-mouse Immunoglobulins HRP	DAKO (P0447)	AB_2617137	1:4000
Polyclonal rabbit anti-goat Immunoglobulins HRP	DAKO (P0449)	AB_26177143	1:4000
Polyclonal rabbit anti-rat Immunoglobulin HRP	DAKO (P0450)	AB_2630354	1:4000
Polyclonal goat anti-rabbit Immunoglobulin HRP	DAKO (P0448)	AB_2617138	1:4000

Supplementary Table 3. HSPs identified in the proteome of a DFTD cell line

Gene symbol*	Name*	Number of unique peptides
**HSP70 superfamily: HSPA (HSP70) and HSPH (HSP110) families**		
HSPA Family		
HSPA2	Heat shock protein family A (Hsp70) member 2	17
HSPA4	Heat shock protein family A (Hsp70) member 4	20
HSPA5	Heat shock protein family A (Hsp70) member 5	33
HSPA8	Heat shock protein family A (Hsp70) member 8	37
HSPA9	Heat shock protein family A (Hsp70) member 9	28
HSPH Family		
HSPH1	Heat shock protein family H (Hsp110) member 1	28
**HSP90 family**		
HSP90AA1	Heat shock protein 90 alpha family class A member 1	24
HSP90AB1	Heat shock protein 90 alpha family class B member 1	40
HSP90B1	Heat shock protein 90 beta family member 1	44
**Chaperonins family**		
HSPD1	Heat shock protein family D (Hsp60) member 1	35
HSPE1	Heat shock protein family E (Hsp10) member 1	5
CCT2	Chaperonin containing TCP1 subunit 2	29
CCT3	Chaperonin containing TCP1 subunit 3	21
CCT4	Chaperonin containing TCP1 subunit 4	20
CCT5	Chaperonin containing TCP1 subunit 5	19
CCT6B	CCT6B	9
CCT7	Chaperonin containing TCP1 subunit 7	15
CCT8	Chaperonin containing TCP1 subunit 8	25
**DNAJ (HSP40) family**		
DNAJA1	DnaJ heat shock protein family (Hsp40) member A1	4
DNAJA2	DnaJ heat shock protein family (Hsp40) member A2	9
DNAJA3	DnaJ heat shock protein family (Hsp40) member A3	2
DNAJB11	DnaJ heat shock protein family (Hsp40) member B11	5
DNAJC8	DnaJ heat shock protein family (Hsp40) member C8	2

*Nomenclature according to the HUGO Gene Nomenclature Committee (HGNC).

Supplementary Table 4. Biological relevance of selected cognate DFTD antigens identified by mass spectrometry analyses

Predicted protein

14-3-3 protein. Spots 7,8,9,10

Function:

Contributes to regulate cell cycle, cell growth, differentiation, survival, apoptosis, migration and spreading.

Associations to cancer:

14-3-3 protein is involved in human gastric cancer cell progression and potential diagnostic and prognostic biomarker in liver and renal cancer. 14-3-3zeta protein is currently undergoing extensive investigation as a novel therapeutic target. 14-3-3 theta protein has been identified as an antigen that induces a humoral response in lung cancer.

References:

(1-9).

60 kDa heat shock protein (HSP60), mitochondrial. Spot 1

Function:

Protein mostly localized in the mitochondrial matrix and outer mitochondrial membrane, constitutively expressed under normal condition, and induced by heat shock, mitochondrial damage, and mitochondrial DNA depletion.

Associations to cancer:

Overexpression reported in various cancers such as adrenal tumours and human breast, large bowel, bronchial, exocervical, ovarian, gastric and prostate cancers. HSP60 is actively secreted by tumour cells and plays a role in transformation, promotion of angiogenesis and metastases. Serum antibodies against HSP60 are elevated in patients with osteosarcoma.

References:

(10-22).

ATP synthase subunit beta, mitochondrial. Spots 4,18

Function:

Enzyme that catalyses ATP synthesis. It also exists outside the cell membrane.

Associations to cancer:

Ectopic (outside the cell membrane) the enzyme has been proposed as a marker for tumour target therapy. The down-regulation of the catalytic subunit of the enzyme is a hallmark of most human carcinomas.

References:

(23-27).

Predicted protein

Cathepsin B. Spot 12

Function:

The protein is a lysosomal cysteine proteinase. It is also known as amyloid precursor protein secretase and is involved in the proteolytic processing of amyloid precursor protein (APP).

Associations to cancer:

The expression and subcellular localization of cathepsins changes during cancer progression and cathepsins are involved in various aspects of tumourigenesis including metastasis and aggressive behaviour. Cathepsin B has been proposed as potential biomarker and therapeutic target in human cancers such as breast, human pancreatic ductal adenocarcinoma (PDA), cervical, colon, endometrial and pancreatic cancers.

References:

(28-35)

Cellular retinoic acid-binding protein 1. Spot 11

Function:

Specific binding protein for a vitamin A and is thought to play an important role in retinoic acid-mediated differentiation and proliferation processes.

Associations to cancer:

It has been found overexpressed in ovarian carcinoma tissues.

References:

(36-39) http://www.ncbi.nlm.nih.gov/gene/1381

Heterogeneous nuclear ribonucleoprotein. Spot 1

Function:

Transcription factor and has a role during cell cycle progression.

Associations to cancer:

The protein has been implicated in tumourigenesis. It was found overexpressed in melanoma and colorectal, oral, lung, nasopharyngeal, pancreatic, prostate and liver cancers.

References:

(40-46).

Perilipin-3. Spot 18

Function:

Protein required for endosome-to-Golgi transport.

Associations to cancer:

Protein strongly expressed in invasive tumours and in lymph node metastasis in cervical dysplasia and invasive carcinoma.

References:

(47-49)

Predicted protein

Rho GDP-dissociation inhibitor 1. Spot 10

Function:

Down-regulator of Rho family GTPases. It prevents nucleotide exchange and membrane association.

Associations to cancer:

The expression of the protein is altered in a variety of cancers including oral squamous cell carcinoma and colorectal cancer. Overexpression of the protein promotes cell motility and lymph node metastasis. Higher frequency of autoantibodies against Rho-GDP proteins was found in nasopharyngeal and acute leukaemia patients.

References:

(50-56).

Stathmin. Spot 16

Function:

Stathmin is a member of a family of microtubule-destabilizing proteins that regulate the dynamics of microtubule polymerization and depolymerisation.

Associations to cancer:

Stathmin is overexpressed across a broad range of human malignancies including leukaemia, lymphoma, neuroblastoma, ovarian, prostatic, breast and lung cancers and mesothelioma. Stathmin is a potential target in cancer therapies that disrupt the mitotic apparatus.

Stathmin is also upregulated in normally proliferating cell lines. In normal cells, stathmin is upregulted in neurons and anterior pituitary cells. In glial cells, stathmin is a constituent of the myelin Sheath.

References:

(57-66)

Tubulin. Spots 4,16,18

Function:

Tubulin is an integral component of microtubules. It occurs mostly as soluble heterodimers consisting primarily of α- and β-tubulin isoforms or as assembled tubulin polymers that form microtubules.

Associations to cancer:

Autoantibodies against tubulin-alpha and tubulin-beta were detected in sera of renal and oral cell carcinoma and chronic myeloid leukaemia patients.

References:

(67-72).

Predicted protein

Vimentin. Spots 1,3,4,5,6,18

Function

Vimentin is a major constituent of the intermediate filament family of proteins. It is ubiquitously expressed in normal mesenchymal cells; it helps maintaining cellular integrity and provides resistance against stress.

Associations to cancer:

Vimentin is overexpressed in various epithelial cancers, including prostate cancer, gastrointestinal tumours, tumours of the central nervous system, breast cancer, malignant melanoma, and lung cancer. Overexpression in cancer correlates well with accelerated tumour growth, invasion, and poor prognosis. Autoantibodies against vimentin were detected in sera from patients with pancreatic cancer. Anti-vimentin therapeutic approaches have also been proposed.

References:

(73-77).

Supplementary Figure 1

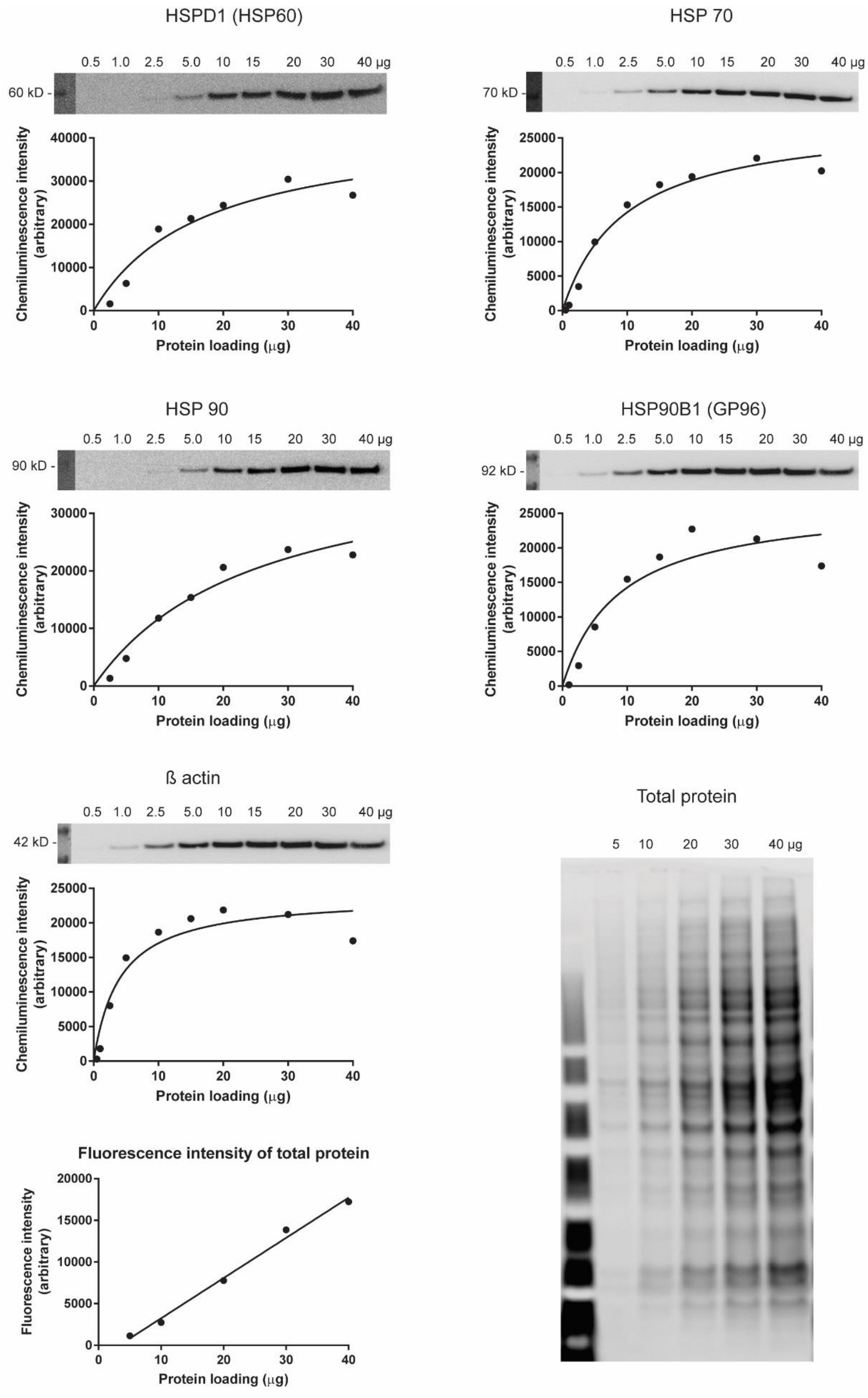

References

1. Mhawech P. 14-3-3 proteins--an update. Cell Res. 2005;15(4):228-36.
2. Tseng CW, Yang JC, Chen CN, Huang HC, Chuang KN, Lin CC, et al. Identification of 14-3-3beta in human gastric cancer cells and its potency as a diagnostic and prognostic biomarker. Proteomics. 2011;11(12):2423-39.
3. Fu H, Subramanian RR, Masters SC. 14-3-3 proteins: structure, function, and regulation. Annu Rev Pharmacol Toxicol. 2000;40:617-47.
4. Pereira-Faca SR, Kuick R, Puravs E, Zhang Q, Krasnoselsky AL, Phanstiel D, et al. Identification of 14-3-3 theta as an antigen that induces a humoral response in lung cancer. Cancer Res. 2007;67(24):12000-6.
5. Yang X, Cao W, Zhang L, Zhang W, Zhang X, Lin H. Targeting 14-3-3zeta in cancer therapy. Cancer Gene Ther. 2011.
6. Zhao J, Meyerkord CL, Du Y, Khuri FR, Fu H. 14-3-3 proteins as potential therapeutic targets. Semin Cell Dev Biol. 2011;22(7):705-12.
7. Freeman AK, Morrison DK. 14-3-3 Proteins: diverse functions in cell proliferation and cancer progression. Semin Cell Dev Biol. 2011;22(7):681-7.
8. Reis H, Putter C, Megger DA, Bracht T, Weber F, Hoffmann AC, et al. A structured proteomic approach identifies 14-3-3Sigma as a novel and reliable protein biomarker in panel based differential diagnostics of liver tumors. Biochimica et biophysica acta. 2015;1854(6):641-50.
9. Kaneko S, Matsumoto K, Minamida S, Hirayama T, Fujita T, Kodera Y, et al. Incremental Expression of 14-3-3 Protein Beta/Alpha in Urine Correlates with Advanced Stage and Poor Survival in Patients with Clear Cell Renal Cell Carcinoma. Asian Pacific journal of cancer prevention : APJCP. 2016;17(3):1399-404.
10. Hansen JJ, Bross P, Westergaard M, Nielsen MN, Eiberg H, Borglum AD, et al. Genomic structure of the human mitochondrial chaperonin genes: HSP60 and HSP10 are localised head to head on chromosome 2 separated by a bidirectional promoter. Hum Genet. 2003;112(1):71-7.
11. Faried A, Sohda M, Nakajima M, Miyazaki T, Kato H, Kuwano H. Expression of heat-shock protein Hsp60 correlated with the apoptotic index and patient prognosis in human oesophageal squamous cell carcinoma. Eur J Cancer. 2004;40(18):2804-11.
12. Ciocca DR, Calderwood SK. Heat shock proteins in cancer: diagnostic, prognostic, predictive, and treatment implications. Cell Stress Chaperones. 2005;10(2):86-103.
13. Lebret T, Watson RW, Molinie V, O'Neill A, Gabriel C, Fitzpatrick JM, et al. Heat shock proteins HSP27, HSP60, HSP70, and HSP90: expression in bladder carcinoma. Cancer. 2003;98(5):970-7.
14. Cappello F. HSP60 and HSP10 as diagnostic and prognostic tools in the management of exocervical carcinoma. Gynecol Oncol. 2003;91(3):661.
15. Cappello F, Rappa F, David S, Anzalone R, Zummo G. Immunohistochemical evaluation of PCNA, p53, HSP60, HSP10 and MUC-2 presence and expression in prostate carcinogenesis. Anticancer Res. 2003;23(2B):1325-31.
16. Cappello F, Bellafiore M, Palma A, David S, Marciano V, Bartolotta T, et al. 60KDa chaperonin (HSP60) is over-expressed during colorectal carcinogenesis. Eur J Histochem. 2003;47(2):105-10.
17. Pignatelli D, Ferreira J, Soares P, Costa MJ, Magalhaes MC. Immunohistochemical study of heat shock proteins 27, 60 and 70 in the normal human adrenal and in adrenal tumors with suppressed ACTH production. Microsc Res Tech. 2003;61(3):315-23.
18. Trieb K, Gerth R, Windhager R, Grohs JG, Holzer G, Berger P, et al. Serum antibodies against the heat shock protein 60 are elevated in patients with osteosarcoma. Immunobiology. 2000;201(3-4):368-76.

19. Wu C, Luo Z, Chen X, Yao D, Zhao P, Liu L, et al. Two-dimensional differential in-gel electrophoresis for identification of gastric cancer-specific protein markers. Oncol Rep. 2009;21(6):1429-37.
20. Tong WW, Tong GH, Kong H, Liu Y. The tumor promoting roles of HSP60 and HIF2alpha in gastric cancer cells. Tumour biology : the journal of the International Society for Oncodevelopmental Biology and Medicine. 2016;37(7):9849-54.
21. Agababaoglu I, Onen A, Demir AB, Aktas S, Altun Z, Ersoz H, et al. Chaperonin (HSP60) and annexin-2 are candidate biomarkers for non-small cell lung carcinoma. Medicine. 2017;96(6):e5903.
22. Wu J, Liu T, Rios Z, Mei Q, Lin X, Cao S. Heat Shock Proteins and Cancer. Trends in pharmacological sciences. 2017;38(3):226-56.
23. Ma Z, Cao M, Liu Y, He Y, Wang Y, Yang C, et al. Mitochondrial F1Fo-ATP synthase translocates to cell surface in hepatocytes and has high activity in tumor-like acidic and hypoxic environment. Acta Biochim Biophys Sin (Shanghai). 2010;42(8):530-7.
24. Lopez-Rios F, Sanchez-Arago M, Garcia-Garcia E, Ortega AD, Berrendero JR, Pozo-Rodriguez F, et al. Loss of the mitochondrial bioenergetic capacity underlies the glucose avidity of carcinomas. Cancer Res. 2007;67(19):9013-7.
25. Cuezva JM, Chen G, Alonso AM, Isidoro A, Misek DE, Hanash SM, et al. The bioenergetic signature of lung adenocarcinomas is a molecular marker of cancer diagnosis and prognosis. Carcinogenesis. 2004;25(7):1157-63.
26. Willers IM, Isidoro A, Ortega AD, Fernandez PL, Cuezva JM. Selective inhibition of beta-F1-ATPase mRNA translation in human tumours. Biochem J. 2010;426(3):319-26.
27. Li W, Li Y, Li G, Zhou Z, Chang X, Xia Y, et al. Ectopic expression of the ATP synthase beta subunit on the membrane of PC-3M cells supports its potential role in prostate cancer metastasis. International journal of oncology. 2017;50(4):1312-20.
28. Gopinathan A, Denicola GM, Frese KK, Cook N, Karreth FA, Mayerle J, et al. Cathepsin B promotes the progression of pancreatic ductal adenocarcinoma in mice. Gut. 2011.
29. Wu D, Wang H, Li Z, Wang L, Zheng F, Jiang J, et al. Cathepsin B may be a potential biomarker in cervical cancer. Histol Histopathol. 2012;27(1):79-87.
30. Chan AT, Baba Y, Shima K, Nosho K, Chung DC, Hung KE, et al. Cathepsin B expression and survival in colon cancer: implications for molecular detection of neoplasia. Cancer Epidemiol Biomarkers Prev. 2010;19(11):2777-85.
31. Watson CJ, Kreuzaler PA. The role of cathepsins in involution and breast cancer. J Mammary Gland Biol Neoplasia. 2009;14(2):171-9.
32. Devetzi M, Scorilas A, Tsiambas E, Sameni M, Fotiou S, Sloane BF, et al. Cathepsin B protein levels in endometrial cancer: Potential value as a tumour biomarker. Gynecol Oncol. 2009;112(3):531-6.
33. Yang WE, Ho CC, Yang SF, Lin SH, Yeh KT, Lin CW, et al. Cathepsin B Expression and the Correlation with Clinical Aspects of Oral Squamous Cell Carcinoma. PLoS One. 2016;11(3):e0152165.
34. Ruan J, Zheng H, Rong X, Rong X, Zhang J, Fang W, et al. Over-expression of cathepsin B in hepatocellular carcinomas predicts poor prognosis of HCC patients. Molecular cancer. 2016;15:17.
35. Swisher LZ, Prior AM, Gunaratna MJ, Shishido S, Madiyar F, Nguyen TA, et al. Quantitative electrochemical detection of cathepsin B activity in breast cancer cell lysates using carbon nanofiber nanoelectrode arrays toward identification of cancer formation. Nanomedicine : nanotechnology, biology, and medicine. 2015;11(7):1695-704.
36. Hibbs K, Skubitz KM, Pambuccian SE, Casey RC, Burleson KM, Oegema TR, Jr., et al. Differential gene expression in ovarian carcinoma: identification of potential biomarkers. Am J Pathol. 2004;165(2):397-414.
37. Turhani D, Krapfenbauer K, Thurnher D, Langen H, Fountoulakis M. Identification of differentially expressed, tumor-associated proteins in oral squamous cell carcinoma by proteomic analysis. Electrophoresis. 2006;27(7):1417-23.

38. Kainov Y, Favorskaya I, Delektorskaya V, Chemeris G, Komelkov A, Zhuravskaya A, et al. CRABP1 provides high malignancy of transformed mesenchymal cells and contributes to the pathogenesis of mesenchymal and neuroendocrine tumors. Cell cycle (Georgetown, Tex). 2014;13(10):1530-9.
39. Favorskaya I, Kainov Y, Chemeris G, Komelkov A, Zborovskaya I, Tchevkina E. Expression and clinical significance of CRABP1 and CRABP2 in non-small cell lung cancer. Tumour biology : the journal of the International Society for Oncodevelopmental Biology and Medicine. 2014;35(10):10295-300.
40. Michelotti EF, Michelotti GA, Aronsohn AI, Levens D. Heterogeneous nuclear ribonucleoprotein K is a transcription factor. Mol Cell Biol. 1996;16(5):2350-60.
41. Carpenter B, McKay M, Dundas SR, Lawrie LC, Telfer C, Murray GI. Heterogeneous nuclear ribonucleoprotein K is over expressed, aberrantly localised and is associated with poor prognosis in colorectal cancer. Br J Cancer. 2006;95(7):921-7.
42. Roychoudhury P, Chaudhuri K. Evidence for heterogeneous nuclear ribonucleoprotein K overexpression in oral squamous cell carcinoma. Br J Cancer. 2007;97(4):574-5; author reply 6.
43. Barboro P, Repaci E, Rubagotti A, Salvi S, Boccardo S, Spina B, et al. Heterogeneous nuclear ribonucleoprotein K: altered pattern of expression associated with diagnosis and prognosis of prostate cancer. Br J Cancer. 2009;100(10):1608-16.
44. Zhou R, Shanas R, Nelson MA, Bhattacharyya A, Shi J. Increased expression of the heterogeneous nuclear ribonucleoprotein K in pancreatic cancer and its association with the mutant p53. Int J Cancer. 2010;126(2):395-404.
45. Du Q, Wang L, Zhu H, Zhang S, Xu L, Zheng W, et al. The role of heterogeneous nuclear ribonucleoprotein K in the progression of chronic myeloid leukemia. Med Oncol. 2010;27(3):673-9.
46. Liu X, Zhou Y, Lou Y, Zhong H. Knockdown of HNRNPA1 inhibits lung adenocarcinoma cell proliferation through cell cycle arrest at G0/G1 phase. Gene. 2016;576(2 Pt 2):791-7.
47. Szigeti A, Minik O, Hocsak E, Pozsgai E, Boronkai A, Farkas R, et al. Preliminary study of TIP47 as a possible new biomarker of cervical dysplasia and invasive carcinoma. Anticancer Res. 2009;29(2):717-24.
48. Muthusamy K, Halbert G, Roberts F. Immunohistochemical staining for adipophilin, perilipin and TIP47. J Clin Pathol. 2006;59(11):1166-70.
49. Westhoff CC, Mrozinski J, Riedel I, Heid HW, Moll R. Perilipin 1 is a highly specific marker for adipocytic differentiation in sarcomas with intermediate sensitivity. Journal of cancer research and clinical oncology. 2017;143(2):225-32.
50. Xiao ZQ, Chen Y, Yi B, Li MY, Zhang PF, Yi H, et al. Identification of nasopharyngeal carcinoma antigens that induce humoral immune response by proteomic analysis. Proteomics Clin Appl. 2007;1(7):688-98.
51. Cui JW, Li WH, Wang J, Li AL, Li HY, Wang HX, et al. Proteomics-based identification of human acute leukemia antigens that induce humoral immune response. Mol Cell Proteomics. 2005;4(11):1718-24.
52. Zhao Z, Liu XF, Wu HC, Zou SB, Wang JY, Ni PH, et al. Rab5a overexpression promoting ovarian cancer cell proliferation may be associated with APPL1-related epidermal growth factor signaling pathway. Cancer Sci. 2010;101(6):1454-62.
53. Dovas A, Couchman JR. RhoGDI: multiple functions in the regulation of Rho family GTPase activities. Biochem J. 2005;390(Pt 1):1-9.
54. Harding MA, Theodorescu D. RhoGDI signaling provides targets for cancer therapy. Eur J Cancer. 2010;46(7):1252-9.
55. Chiang WF, Ho HC, Chang HY, Chiu CC, Chen YL, Hour TC, et al. Overexpression of Rho GDP-dissociation inhibitor alpha predicts poor survival in oral squamous cell carcinoma. Oral Oncol. 2011;47(6):452-8.

56. Wang H, Wang B, Liao Q, An H, Li W, Jin X, et al. Overexpression of RhoGDI, a novel predictor of distant metastasis, promotes cell proliferation and migration in hepatocellular carcinoma. FEBS letters. 2014;588(3):503-8.
57. Mistry SJ, Bank A, Atweh GF. Targeting stathmin in prostate cancer. Mol Cancer Ther. 2005;4(12):1821-9.
58. Zhang HZ, Wang Y, Gao P, Lin F, Liu L, Yu B, et al. Silencing stathmin gene expression by survivin promoter-driven siRNA vector to reverse malignant phenotype of tumor cells. Cancer Biol Ther. 2006;5(11):1457-61.
59. Cassimeris L. The oncoprotein 18/stathmin family of microtubule destabilizers. Curr Opin Cell Biol. 2002;14(1):18-24.
60. Rubin CI, Atweh GF. The role of stathmin in the regulation of the cell cycle. J Cell Biochem. 2004;93(2):242-50.
61. Ghosh R, Gu G, Tillman E, Yuan J, Wang Y, Fazli L, et al. Increased expression and differential phosphorylation of stathmin may promote prostate cancer progression. Prostate. 2007;67(10):1038-52.
62. Rana S, Maples PB, Senzer N, Nemunaitis J. Stathmin 1: a novel therapeutic target for anticancer activity. Expert Rev Anticancer Ther. 2008;8(9):1461-70.
63. Belletti B, Baldassarre G. Stathmin: a protein with many tasks. New biomarker and potential target in cancer. Expert Opin Ther Targets. 2011;15(11):1249-66.
64. Gould RM, Oakley T, Goldstone JV, Dugas JC, Brady ST, Gow A. Myelin sheaths are formed with proteins that originated in vertebrate lineages. Neuron Glia Biol. 2008;4(2):137-52.
65. Alesi GN, Jin L, Li D, Magliocca KR, Kang Y, Chen ZG, et al. RSK2 signals through stathmin to promote microtubule dynamics and tumor metastasis. Oncogene. 2016;35(41):5412-21.
66. Reyes HD, Miecznikowski J, Gonzalez-Bosquet J, Devor EJ, Zhang Y, Thiel KW, et al. High stathmin expression is a marker for poor clinical outcome in endometrial cancer: An NRG oncology group/gynecologic oncology group study. Gynecologic oncology. 2017;146(2):247-53.
67. Luduena RF. Multiple forms of tubulin: different gene products and covalent modifications. Int Rev Cytol. 1998;178:207-75.
68. Prasannan L, Misek DE, Hinderer R, Michon J, Geiger JD, Hanash SM. Identification of beta-tubulin isoforms as tumor antigens in neuroblastoma. Clin Cancer Res. 2000;6(10):3949-56.
69. Kellner R, Lichtenfels R, Atkins D, Bukur J, Ackermann A, Beck J, et al. Targeting of tumor associated antigens in renal cell carcinoma using proteome-based analysis and their clinical significance. Proteomics. 2002;2(12):1743-51.
70. Zou L, Wu Y, Pei L, Zhong D, Gen M, Zhao T, et al. Identification of leukemia-associated antigens in chronic myeloid leukemia by proteomic analysis. Leuk Res. 2005;29(12):1387-91.
71. Shukla S, Govekar RB, Sirdeshmukh R, Sundaram CS, D'Cruz AK, Pathak KA, et al. Tumor antigens eliciting autoantibody response in cancer of gingivo-buccal complex. Proteomics Clin Appl. 2007;1(12):1592-604.
72. Visconti R, Grieco D. Fighting tubulin-targeting anticancer drug toxicity and resistance. Endocrine-related cancer. 2017;24(9):T107-t17.
73. Hong SH, Misek DE, Wang H, Puravs E, Hinderer R, Giordano TJ, et al. Identification of a Specific Vimentin Isoform That Induces an Antibody Response in Pancreatic Cancer. Biomark Insights. 2006;1:175-83.
74. Lahat G, Zhu QS, Huang KL, Wang S, Bolshakov S, Liu J, et al. Vimentin is a novel anti-cancer therapeutic target; insights from in vitro and in vivo mice xenograft studies. PLoS One. 2010;5(4):e10105.
75. Kokkinos MI, Wafai R, Wong MK, Newgreen DF, Thompson EW, Waltham M. Vimentin and epithelial-mesenchymal transition in human breast cancer--observations in vitro and in vivo. Cells Tissues Organs. 2007;185(1-3):191-203.
76. Satelli A, Li S. Vimentin in cancer and its potential as a molecular target for cancer therapy. Cell Mol Life Sci. 2011;68(18):3033-46.

77. Dmello C, Sawant S, Alam H, Gangadaran P, Mogre S, Tiwari R, et al. Vimentin regulates differentiation switch via modulation of keratin 14 levels and their expression together correlates with poor prognosis in oral cancer patients. PLoS One. 2017;12(2):e0172559.
