# Supplementary material for: Heat shock proteins expressed in the marsupial Tasmanian devil are potential antigenic candidates in a vaccine against devil facial tumour disease
Source: PLoS One. 2018 Apr 27;13(4):e0196469. doi: 10.1371/journal.pone.0196469 (PMC5922574; doi:10.1371/journal.pone.0196469)
Supplement: S2 Table — (PDF) [file pone.0196469.s006.pdf]

S2 Table. Antibodies used in the study

Antibody	Supplier	Antibody Registry number	Dilution
Polyclonal goat anti HSP60	Abcam (Ab82520)	AB_1658326	1:500
Monoclonal mouse anti HSP70	Abcam (Ab2787)	AB_303300	1:1000
Polyclonal rabbit anti HSP90	Abcam (Ab13495)	AB_1269122	1:1000
Monoclonal rat anti Grp94	Abcam (Ab90458)	AB_2295611	1:1000
Polyclonal rabbit anti β-actin	Abcam (Ab8227)	AB_2305186	1:1000
Polyclonal goat anti-mouse Immunoglobulins HRP	DAKO (P0447)	AB_2617137	1:4000
Polyclonal rabbit anti-goat Immunoglobulins HRP	DAKO (P0449)	AB_26177143	1:4000
Polyclonal rabbit anti-rat Immunoglobulin HRP	DAKO (P0450)	AB_2630354	1:4000
Polyclonal goat anti-rabbit Immunoglobulin HRP	DAKO (P0448)	AB_2617138	1:4000
