# Supplementary material for: Heat shock proteins expressed in the marsupial Tasmanian devil are potential antigenic candidates in a vaccine against devil facial tumour disease
Source: PLoS One. 2018 Apr 27;13(4):e0196469. doi: 10.1371/journal.pone.0196469 (PMC5922574; doi:10.1371/journal.pone.0196469)
Supplement: S3 Table — (PDF) [file pone.0196469.s007.pdf]

S3 Table. HSPs identified in the proteome of a DFTD cell line (1)

Gene symbol*	Name*	Number of unique peptides
**HSP70 superfamily: HSPA (HSP70) and HSPH (HSP110) families**		
HSPA Family		
HSPA2	Heat shock protein family A (Hsp70) member 2	17
HSPA4	Heat shock protein family A (Hsp70) member 4	20
HSPA5	Heat shock protein family A (Hsp70) member 5	33
HSPA8	Heat shock protein family A (Hsp70) member 8	37
HSPA9	Heat shock protein family A (Hsp70) member 9	28
HSPH Family		
HSPH1	Heat shock protein family H (Hsp110) member 1	28
**HSP90 family**		
HSP90AA1	Heat shock protein 90 alpha family class A member 1	24
HSP90AB1	Heat shock protein 90 alpha family class B member 1	40
HSP90B1	Heat shock protein 90 beta family member 1	44
**Chaperonins family**		
HSPD1	Heat shock protein family D (Hsp60) member 1	35
HSPE1	Heat shock protein family E (Hsp10) member 1	5
CCT2	Chaperonin containing TCP1 subunit 2	29
CCT3	Chaperonin containing TCP1 subunit 3	21
CCT4	Chaperonin containing TCP1 subunit 4	20
CCT5	Chaperonin containing TCP1 subunit 5	19
CCT6B	CCT6B	9
CCT7	Chaperonin containing TCP1 subunit 7	15
CCT8	Chaperonin containing TCP1 subunit 8	25
**DNAJ (HSP40) family**		
DNAJA1	DnaJ heat shock protein family (Hsp40) member A1	4
DNAJA2	DnaJ heat shock protein family (Hsp40) member A2	9
DNAJA3	DnaJ heat shock protein family (Hsp40) member A3	2
DNAJB11	DnaJ heat shock protein family (Hsp40) member B11	5
DNAJC8	DnaJ heat shock protein family (Hsp40) member C8	2

*Nomenclature according to the HUGO Gene Nomenclature Committee (HGNC).

1. Patchett AL, Wilson R, Charlesworth JC, Corcoran LM, Papenfuss AT, Lyons AB, et al. Transcriptome and proteome profiling reveals stress-induced expression signatures of imiquimod-treated Tasmanian devil facial tumor disease (DFTD) cells. Oncotarget. 2018;9:15895-914.
